# OMIP-101: 27-color flow cytometry panel for immunophenotyping of major leukocyte populations in fixed whole blood

**DOI:** 10.1002/cyto.a.24827

**Published:** 2024-02-11

**Authors:** Claire Imbratta, Tim Reid, Asma Toefy, Thomas J. Scriba, Elisa Nemes

**Affiliations:** 1 South African Tuberculosis Vaccine Initiative, Division of Immunology, Department of Pathology, Institute of Infectious Disease and Molecular Medicine, University of Cape Town, Cape Town, South Africa

**Keywords:** Immunophenotyping, high-dimensional flow cytometry, absolute counts, fixed whole blood, clinical trials, immune cell subsets

## Abstract

This 27-color flow cytometry antibody panel allows broad immune-profiling of major leukocyte subsets in human whole blood (WB). It includes lineage markers to identify myeloid and lymphoid cell populations including granulocytes, monocytes, myeloid dendritic cells (mDCs), natural killer (NK) cells, NKT-like cells, B cells, conventional CD4 and CD8 T cells, γδ T cells, mucosa-associated invariant T (MAIT) cells and innate lymphoid cells (ILC). To further characterize each of these populations, markers defining stages of cell differentiation (CCR7, CD27, CD45RA, CD127, CD57), cytotoxic potential (perforin, granzyme B) and cell activation/proliferation (HLA-DR, CD38, Ki-67) were included. This panel was developed for quantifying absolute counts and phenotyping major leukocyte populations in cryopreserved, fixed WB collected from participants enrolled in large multi-site tuberculosis (TB) vaccine clinical trials (Table 1). This antibody panel can be applied to profile major leukocyte subsets in other sample types such as fresh WB or peripheral blood mononuclear cells (PBMCs) with only minor additional optimization.

## 1. Background

**Figure 1.**
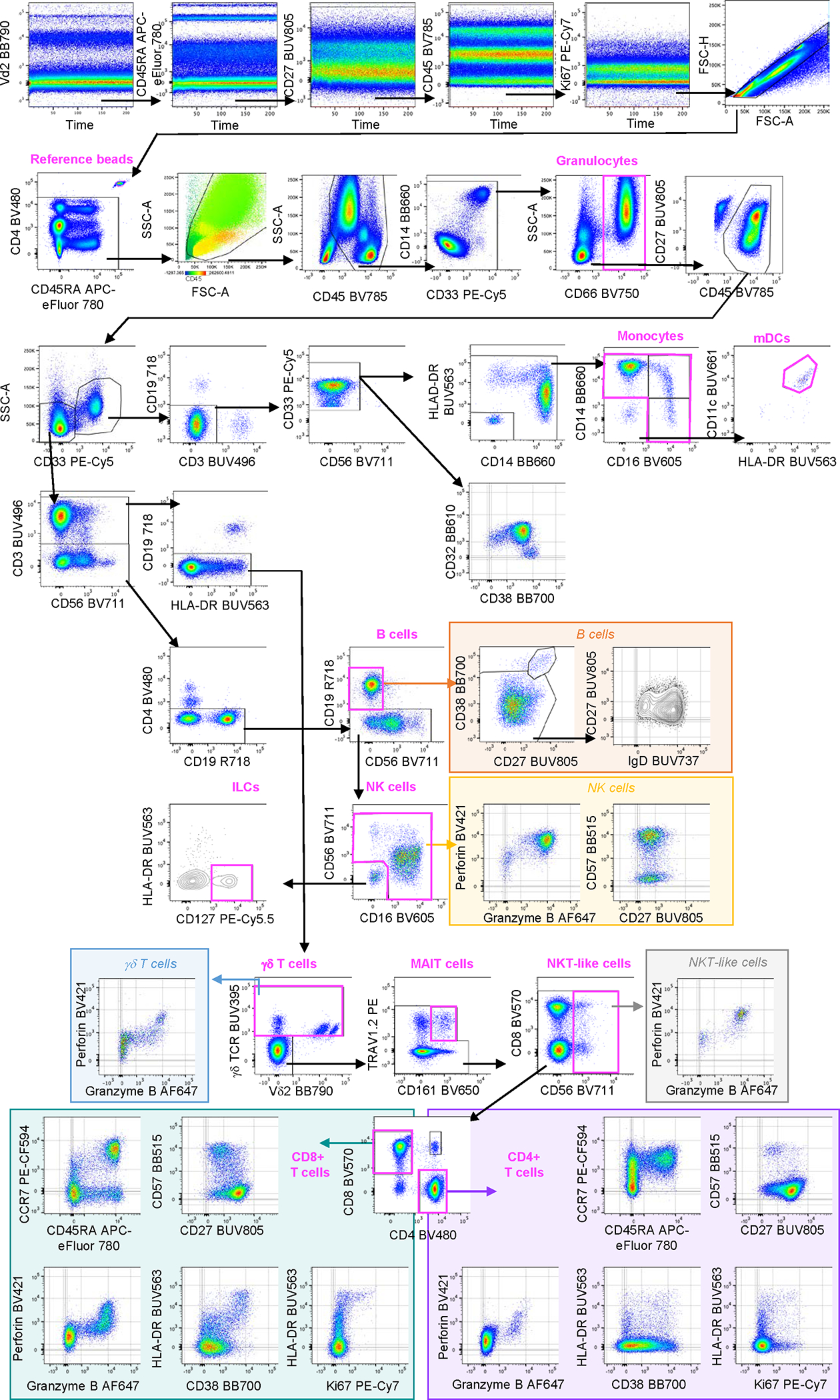
Hierarchical gating strategy Representative example of manual gating strategy for fixed whole blood using BD FACSymphony A5 cytometer B. Labels in bold above graphs are used to indicate cell lineages / reference beads. Time gates were applied to the detector measuring the longer wavelength off each of the lasers, to exclude any inconsistent fluorescence patterns during sample acquisition. After exclusion of doublets, aggregates and reference beads, leukocytes were firstly selected based on SSC/FSC overlaid with CD45 and refined using CD45 and SSC parameters. Antibody aggregates were identified as extremes by plotting CD14 vs CD33 (the fluorochrome combination that most frequently allowed visualization of aggregates) and excluded. Following exclusion of CD66+ granulocytes and additional cleaning, CD66− myeloid and lymphoid cell populations were delineated based on CD33 expression. From the CD33+ myeloid gate, CD19+, CD3+ and CD56+ cells were excluded and HLA-DR+ cells were selected. Monocytes were stratified based on CD16/CD14 markers into classical (CD14+CD16−), intermediate (CD14+CD16+/low) and non-classical (CD14-CD16+) monocytes. From the double negative population (CD14-CD16−), mDCs were identified as HLA-DR+CD11c+. Expression of CD32 and CD38 was assessed on all CD33+ myeloid cells. From the lymphocyte (CD33− and SSC low) gate, B cells were defined as CD3−, CD4−, CD56− and CD19+ cells. Plasmablasts (CD38 and CD27 high) were identified among CD19+ B cells. B cells were further classified into naïve (IgD+CD27−) and memory (IgD-CD27+) populations. NK cells were identified after exclusion of CD3, CD4 and CD19 based on CD56 (dim and bright) and CD16 (positive or negative) expression. Overlay of perforin expression was visualized to confirm that CD56dim CD16+ cells were NK cells (not shown). Their cytotoxic and memory patterns were evaluated using perforin/granzyme B and CD57/CD27, respectively. CD3−, CD4−, CD19−, CD16−, CD56− lymphocytes expressing CD127 were categorized as ILCs. From the lymphocyte (CD33− and SSC low) gate, T cells were gated based on CD3 expression followed by exclusion of CD19+ B cells. γδ TCR+ T cells were further classified based on Vδ2 expression. Inclusion of perforin and granzyme B allowed evaluation of their cytotoxic potential. From the γδ TCR− T cell gate, phenotypic MAIT cells were identified based on TRAV1.2 and CD161 co-expression. Following exclusion of this lineage, NKT-like cells (CD56+) and conventional T cells (CD56−) were selected. Cytotoxic potential of CD3+CD56+ NKT-like cells was further characterized by perforin and granzyme B expression. Conventional T cells were then classified into the following populations: single positive CD4 T cells (CD4+CD8−), single positive CD8 T cells (CD4−CD8+) and double positives (CD4+CD8+). CCR7 and CD45RA were further used to stratify CD8 and CD4 T cells into naïve (CCR7+CD45RA+), central memory (CCR7+CD45RA−), effector memory (CCR7-CD45RA−) and terminally differentiated effector memory (CCR7-CD45RA+) cells. CD57, CD127 (not shown) and CD27 were also used to refine differentiation/memory subsets. Expression of proliferation (Ki67), cytotoxic (perforin, granzyme B) and activation (HLA-DR and CD38) markers was also assessed on CD4 and CD8 conventional T cells.

We developed this antibody panel in the context of a research program that aims to identify vaccine-induced immune correlates of protection against *Mycobacterium tuberculosis* infection or TB disease (1). The panel was designed to enumerate and phenotype relevant leukocyte subsets that may be modulated by vaccination in fixed whole blood samples collected from participants enrolled in large TB vaccine clinical trials. We applied a procedure termed Differential Leukocyte Counting and Immunophenotyping in Cryopreserved Ex vivo whole blood (DLC-ICE) (2), based on erythrocyte lysis and cell fixation of a precise volume of blood, prior to cryopreservation. After thawing in batches, fixed cells are permeabilized and stained with the antibody panel, and reference fluorescent microbeads are added to the final cell suspension to accurately determine absolute cell counts by flow cytometry. Use of cryopreserved fixed whole blood in this assay offers multiple benefits compared to other flow cytometry-based methods, including reduction of procedural complexity and workload at the point of sample collection, cost-effectiveness, improvement and reduction of data variability, especially in extensive studies involving multiple sites in resource-limited settings. In a previous report, we demonstrated accuracy, robustness, low inter-operator variability of this method and high concordance of readouts including a wide range of absolute counts and frequencies when fresh and cryopreserved fixed WB samples from the same donors were stained with a similar 27-color panel (3). These results suggest that fixation/permeabilization and cryopreservation steps do not affect the detection and resolution of the selected antibody-marker combinations. Markers included in this panel allow broad analysis of most lymphoid populations, while myeloid subsets can also be characterized to a lesser extent, as described below (Figure 1 and Online Figure 1).

Granulocytes are identified by the expression of CD66, a member of the carcinoembryonic antigen (CEA) family and related to the immunoglobulin superfamily of glycoproteins. Also called polymorphonuclear neutrophilic leukocytes (PMNs), granulocytes include three different types of subsets namely, neutrophils, basophils and eosinophils. We selected the anti-CD66 antibody B1.1 clone, which recognises isoforms that are expressed solely by neutrophils in the blood. Phagocytic neutrophils recognize and internalize pathogenic microorganisms by phagocytosis into microbicidal acidified phagolysosomes, where the organisms are killed through release of antimicrobial enzymes stored in their cytoplasmic granules (4).

Myeloid and lymphoid cells can be distinguished using CD33 expression and side scatter. Monocytes can be divided into three populations based on their CD14 and CD16 expression, namely classical (CD14^high^ CD16^−^), intermediate (CD14^int^ CD16^int^) and non-classical (CD14^−^CD16^+^) monocytes. Myeloid DCs, also named conventional DCs (cDCs), are CD14 and CD16 negative and express high levels of CD11c and HLA-DR. Defined as professional antigen-presenting cells, mDCs are crucial for T cell priming. Through their ability to process ingested pathogens and present microbial peptide antigens to T cells while providing essential co-stimulatory signals, they orchestrate T cell activation and differentiation in response to microbial recognition (5). We did not include CD123, which precluded precise identification of plasmacytoid DC (pDCs, CD123^+^ CD11c^−^) with our panel.

We included two Fc receptors (FcRs), CD32 and CD16, in our panel because of the recently described role of Fc-mediated functions in protection against malaria following vaccination (6). As receptors for the Fc portion of immunoglobulins (Ig), FcRs are transmembrane glycoproteins expressed by a variety of leukocytes including granulocytes, monocytes, B and NK cells. FcR cross-linking mediates positive and negative regulation of cellular signalling including phagocytosis, antibody-dependent cellular cytotoxicity (ADCC), production and release of cytokines and pro-inflammatory molecules. Importantly, FcRs can also induce clearance of immune complexes. In fact, following capture and internalization/degradation of antigen-antibody complex, peptide antigens are presented to cells in an MHC-restricted manner. The largest family of Fc receptors are the IgG receptors or FcγRs which themselves contain several groups: FcγRI/CD64, the high affinity IgG receptor, the FcγRII/CD32 family (FcγRIIA, FcγRIIB, FcγRIIC), and the FcγRIII/CD16 family (7).

Amongst the CD3− cells, we were able to distinguish B cells and several stages of their differentiation. Rather than CD20, we decided to stain for CD19, which is also expressed by plasmablasts, a rare B cell subset expressing CD27 and CD38 at high levels. While they can secrete antibodies at high rate, plasmablasts divide and express surface Ig and MHC class II, allowing antigen presentation to T cells. Following division, they either die or undergo differentiation into short or long-lived plasma cells, the latter migrating to bone marrow (8). Plasmablasts are typically absent or detectable at very low frequencies in healthy individuals, but frequencies usually transiently increase after vaccination. Based on CD27 and IgD expression, memory (IgD^−^CD27^+^) and naïve (IgD^+^CD27^−^) B cells can be distinguished. However, even if we observed CD27 staining patterns as expected on T cells, we note that the resolution of CD27 staining was poor on B cells. We recommend testing different CD27 clones and fluorochrome conjugates if CD27 measurement on B cells is an essential desired outcome in other studies. Expression of IgM and IgG isotypes allow class switch identification, but these markers were not included in our panel due to low priority and poor resolution of staining in fixed whole blood, respectively. Similarly, CXCR5, a chemokine receptor for CXCL13 and important soluble factor for germinal center establishment and maintenance, was tested during panel optimisation, but resulted in particularly poor resolution on T cells and therefore was excluded. ILCs were identified using CD127 after exclusion of most lineage markers.

NK cells, defined as CD3^−^CD56^+^CD16^+/−^, are innate cells with the ability to kill infected cells. Their cytolytic granules, containing pore-forming protein perforin and granzymes are released at the surface of the target cell and can penetrate the cell membrane to mediate programmed cell death (9). NK cells can be classified into two main types based on expression of CD56 and CD16: highly cytotoxic CD56^dim^CD16^+^ mature cells and CD56^bri^CD16^−^ immature cells that can produce cytokines, especially IFN-γ, that can polarize CD4 T cells towards Th1 pro-inflammatory cells. In this panel, we included the cytotoxic markers perforin and granzyme B. CD57 and CD27 were included as NK (and T) cell differentiation markers, facilitating the delineation of NK cell maturation from CD56^bri^ to CD56^dim^CD57^+^ cells, which have decreased proliferative but retain highly cytotoxic capacity (10, 11). Of note, in another study, CD57^+^ NKG2C^+^ NK cells revealed memory-like properties specific to acute MCMV infection (12). Similarly, Venkatasubramanian *et al*. suggested CD27 as a potential ‘memory’ marker for BCG-induced memory-like NK cells (13).

Donor-unrestricted T cells (DURT cells) include MR1-restricted MAIT cells, CD1-restricted invariant NKT (iNKT) cells, germline-encoded mycolyl-reactive (GEM) cells, glucose monomycolate-reactive (GMM) cells, and γδ T cells. DURT cells recognize non-peptide antigens such as lipids, vitamin B metabolites or phosphoantigens presented by non-polymorphic MHC-like and non-MHC-like molecules (i.e CD1d for iNKT, MR1 for MAIT, CD1b for GMM cells) (14). While MAIT and CD1-restricted DURT cells predominantly recognize antigens via αβ T cell receptors (TCRs), γδ T cells recognize antigens via γδ TCRs. Vδ2^+^ γδ T cells are the most dominant subset of γδ T cells in adult human blood and can be profiled with this panel (15). DURT cells are optimally defined using tetramers or by detecting specific invariant TCRs. Staining of fixed cells with MR1–5OP-RU and CD1d-αGalCer tetramers, or anti-Vα24Jα18 TCR antibody was suboptimal, yielding very high inter-individual variability (MR1 tetramer) or poor staining resolution (CD1d- tetramer and anti-Vα24Jα18,) and these reagents were not included. Rather, anti-CD161 and anti-TRAV1.2 (Vα7.2) antibodies were used to phenotypically identify MAIT cells, while CD56 expression on T cells was used to identify NKT-like cells. Conventional CD4^+^ and CD8^+^ T cells were identified after exclusion of DURT cell subsets.

We included several markers to characterize lymphocyte differentiation. Expression levels of CCR7 and CD45RA were primarily used to distinguish between memory and naïve T cells and between memory and effector T cell compartments. Four main subsets were identified, namely naïve T cells, central memory T cells (CM), effector memory (EM) and terminally differentiated effector T cells (EMRA). While naïve T cells express both CCR7 and CD45RA, CM lose CD45RA during transition from naïve to memory stage. Following antigen exposure, CM lose CCR7 expression and differentiate into EM and, typically after chronic activation, finally into EMRA, which lack CCR7 and re-express CD45RA (16). CD27, CD57 and CD127 were used to further refine naïve and memory T cell subsets. CD27 is a co-stimulatory receptor important for activation of T cells during the early stages of differentiation. The oligosaccharide CD57 is an end-stage marker associated with T cell senescence and prone to activation-induced cell death. CD127 (IL-7 receptor α) regulates T cell homeostasis though proliferation and survival of memory T cells. As mentioned above, most of these differentiation markers can also be used to characterize lymphocyte subsets other than conventional T cells.

In this panel, we also included HLA-DR and CD38, which serve to differentiate myeloid cell subsets and identify activated T cells, as well as Ki-67, a proliferation marker expressed by all cell types during cell cycle. These activation markers are expressed for several days after *in vivo* upregulation, and are therefore useful to monitor *ex vivo*, non-specific activation of immune cells during infections and following treatment or vaccination (17–19).

To summarize, this panel enables identification and broad characterization of most relevant circulating leukocyte populations, including lymphoid and myeloid cell subsets and their phenotypes in human peripheral blood (Table 2). It may be used to track changes in immune cell subset abundance and expression of surface and intracellular markers that are modulated during different stages of infection and disease, as well as upon treatment, particularly by immunotherapy and vaccination. Importantly, we verified that all antibody clones included in the panel stained live, unfixed cells as well as fixed cells with similar performance. We recommend performing the same validation of individual antibodies and fluorochrome conjugates not reported here.

## 2. Similarity to published OMIPs

Although OMIP-063, -069 and -078 focused on immune-profiling of major immune cell subsets by flow cytometry, these panels were optimized using PBMCs. To our knowledge, few extensive panels focusing on a broad range of leukocytes were developed on whole blood (OMIP-077, -062) and none used fixative prior to staining of extra-cellular markers. In fact, this 27-color panel is the first to enable comprehensive characterization of cellular composition of fixed and cryopreserved whole blood. This is particularly valuable to measure expression of surface and intracellular markers on most cell populations, especially when sample availability is limited, for example in paediatric studies or large clinical studies. Myeloid populations are defined using five of the 27 markers, while we identified lymphoid cells with nine markers. In addition, we included additional markers to allow characterization of *ex vivo* cell differentiation, activation, function and proliferation. HLA-DR, CD11c, CD16, CD32, CD38 and CD45RA can be expressed by both myeloid and lymphoid compartments.

## 3. Human samples

All participants provided written, informed consent and the protocol was approved by the Human Research Ethics Committee of University of Cape Town, South Africa. The study was conducted according to the principles of the Declaration of Helsinki. Venous WB was collected in sodium heparin tubes from healthy, adult donors.

## Supplementary Material

Supinfo1

Supinfo2

## Figures and Tables

**Table 1.** Summary table for application of OMIP-101.

Purpose	Broad immunophenotyping of leukocytes including myeloid (neutrophils, monocytes, mDCs) and lymphoid cell lineages (conventional T cells, DURT cells, γδ T cells, B cells, NK cells, ILCs)
Species	Human
Cell type	Fresh or fixed, cryopreserved whole blood
Cross-references	OMIP-062, -063, -069, -077, -078

**Table 2.** Reagents used in OMIP-101.

Specificity	Fluorochrome	Clone	Purpose	
CD45	BV785	HI30	Leukocytes	Lineage
CD66	BV750	B1.1	Granulocytes	
CD33	PE-Cy5	WM53	Myeloid cells	
CD14	BB660	MφP9	Monocytes	
CD11c	BUV661	B-ly6	Myeloid dendritic cells	
CD19	R718	HIB19	B cells	
CD56	BV711	HCD56	NK cells, NKT-like cells	
CD127	PE-Cy5.5	eBioRD5	ILCs, T cell differentiation	
CD3	BUV496	UCHT-1	T cells	
CD4	BV480	SK3	Helper T cells	
CD8	BV570	RPA-T8	Cytotoxic T cells	
γδ TCR	BUV395	B1	γδ T cells	
TRAV1.2	PE	3C10	MAIT cells	
CD161	BV650	DX12	MAIT cells, T/NK cell differentiation	
CD38	BB700	HIT2	Monocyte, mDC, T cell, and B cell activation/differentiation, plasmablasts	Phenotype / cell subsets
HLA-DR	BUV563	G46-6	Myeloid subsets, T cell activation	
CD32	BB630	FLI8.26	Fc receptor	
CD16	BV605	3G8	Monocyte, NK, and mDC subsets	
IgD	BUV737	IA6-2	B cell differentiation	
CD27	BUV805	M-T271	T, B, and NK cell differentiation	
CD57	BB515	NK-1	NK and T cell differentiation	
CD45RA	APC-eFluor 780	HI100	T cell differentiation	
CCR7	PE-CF594	150503	T cell differentiation	
Vδ2	BB790	B6	γδ T cell subset	
Perforin	BV421	γG9	CD8, NK and NKT cell cytotoxicity	Function
Granzyme B	AF647	DH2	CD8, NK and NKT-like cell cytotoxicity	
Ki67	PE-Cy7	B56	Proliferation	
