# Supplementary material for: OMIP-101: 27-color flow cytometry panel for immunophenotyping of major leukocyte populations in fixed whole blood
Source: Cytometry A. Author manuscript; Available in PMC 2024 Mar 22. (PMC10958279; doi:10.1002/cyto.a.24827)
Supplement: Supinfo1 [file NIHMS1964512-supplement-Supinfo1.pptx]

## Slide 1
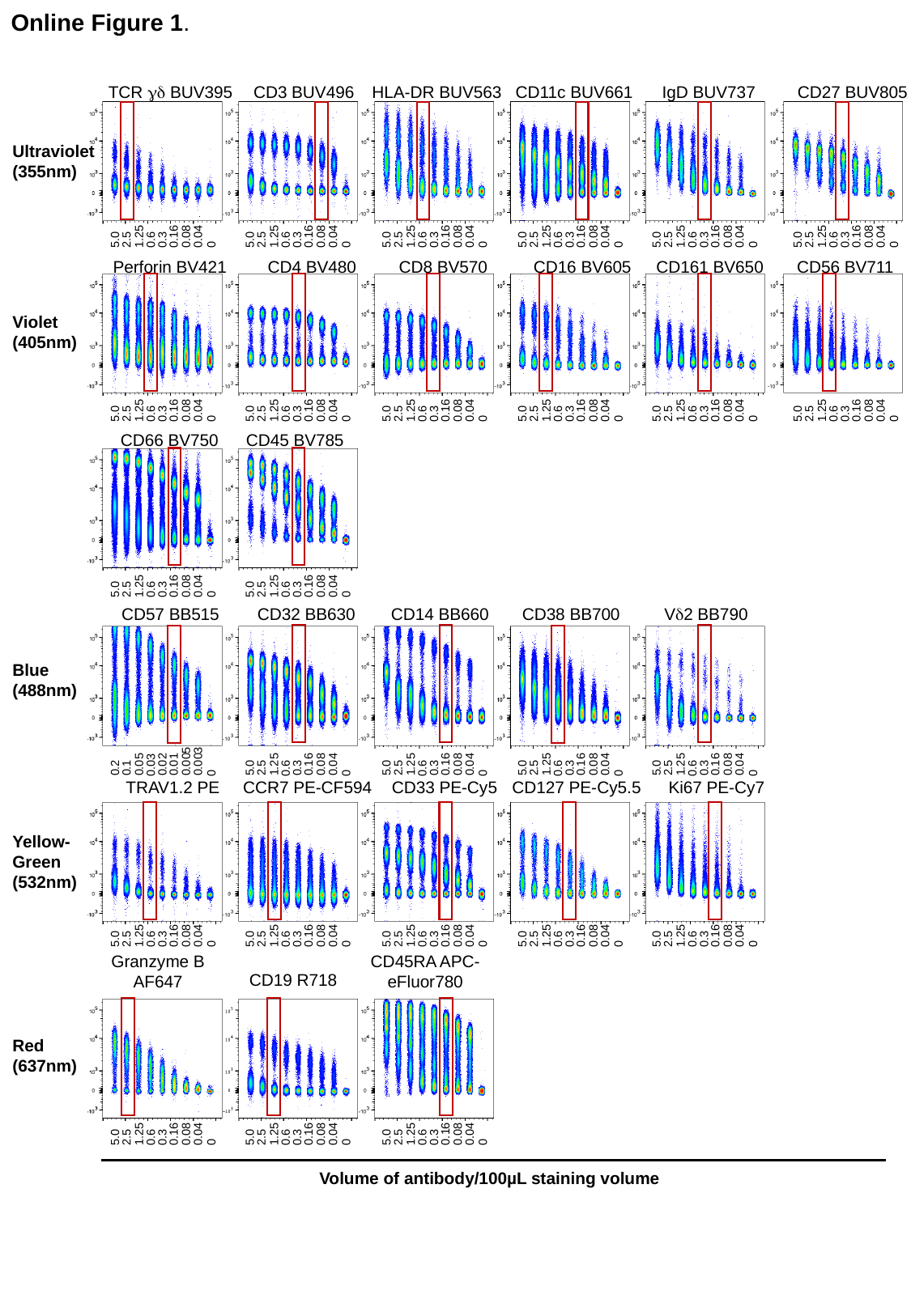

Online Figure 1.
TCR gd BUV395
CD11c BUV661
CD27 BUV805
CD3 BUV496
HLA-DR BUV563
IgD BUV737
Ultraviolet
(355nm)
0.04
0.08
0.6
0.16
1.25
5.0
2.5
0.3
0
0.04
0.08
0.6
0.16
1.25
5.0
2.5
0.3
0
0.04
0.08
0.6
0.16
1.25
5.0
2.5
0.3
0
0.04
0.08
0.6
0.16
1.25
5.0
2.5
0.3
0
0.04
0.08
0.6
0.16
1.25
5.0
2.5
0.3
0
0.04
0.08
0.6
0.16
1.25
5.0
2.5
0.3
0
CD4 BV480
CD161 BV650
CD56 BV711
Perforin BV421
CD8 BV570
CD16 BV605
Violet
(405nm)
0.04
0.08
0.6
0.16
1.25
5.0
2.5
0.3
0
0.04
0.08
0.6
0.16
1.25
5.0
2.5
0.3
0
0.04
0.08
0.6
0.16
1.25
5.0
2.5
0.3
0
0.04
0.08
0.6
0.16
1.25
5.0
2.5
0.3
0
0.04
0.08
0.6
0.16
1.25
5.0
2.5
0.3
0
0.04
0.08
0.6
0.16
1.25
5.0
2.5
0.3
0
CD66 BV750
CD45 BV785
0.04
0.08
0.6
0.16
1.25
5.0
2.5
0.3
0
0.04
0.08
0.6
0.16
1.25
5.0
2.5
0.3
0
CD14 BB660
CD38 BB700
CD57 BB515
CD32 BB630
Vd2 BB790
Blue
(488nm)
0.005
0.003
0.02
0.03
0.01
0.05
0.2
0.1
0
0.04
0.08
0.6
0.16
1.25
5.0
2.5
0.3
0
0.04
0.08
0.6
0.16
1.25
5.0
2.5
0.3
0
0.04
0.08
0.6
0.16
1.25
5.0
2.5
0.3
0
0.04
0.08
0.6
0.16
1.25
5.0
2.5
0.3
0
CD127 PE-Cy5.5
TRAV1.2 PE
CCR7 PE-CF594
CD33 PE-Cy5
Ki67 PE-Cy7
Yellow-Green
(532nm)
0.04
0.08
0.6
0.16
1.25
5.0
2.5
0.3
0
0.04
0.08
0.6
0.16
1.25
5.0
2.5
0.3
0
0.04
0.08
0.6
0.16
1.25
5.0
2.5
0.3
0
0.04
0.08
0.6
0.16
1.25
5.0
2.5
0.3
0
0.04
0.08
0.6
0.16
1.25
5.0
2.5
0.3
0
Granzyme B
AF647
CD45RA APC-eFluor780
CD19 R718
Red
(637nm)
0.04
0.08
0.6
0.16
1.25
5.0
2.5
0.3
0
0.04
0.08
0.6
0.16
1.25
5.0
2.5
0.3
0
0.04
0.08
0.6
0.16
1.25
5.0
2.5
0.3
0
Volume of antibody/100µL staining volume

## Slide 2
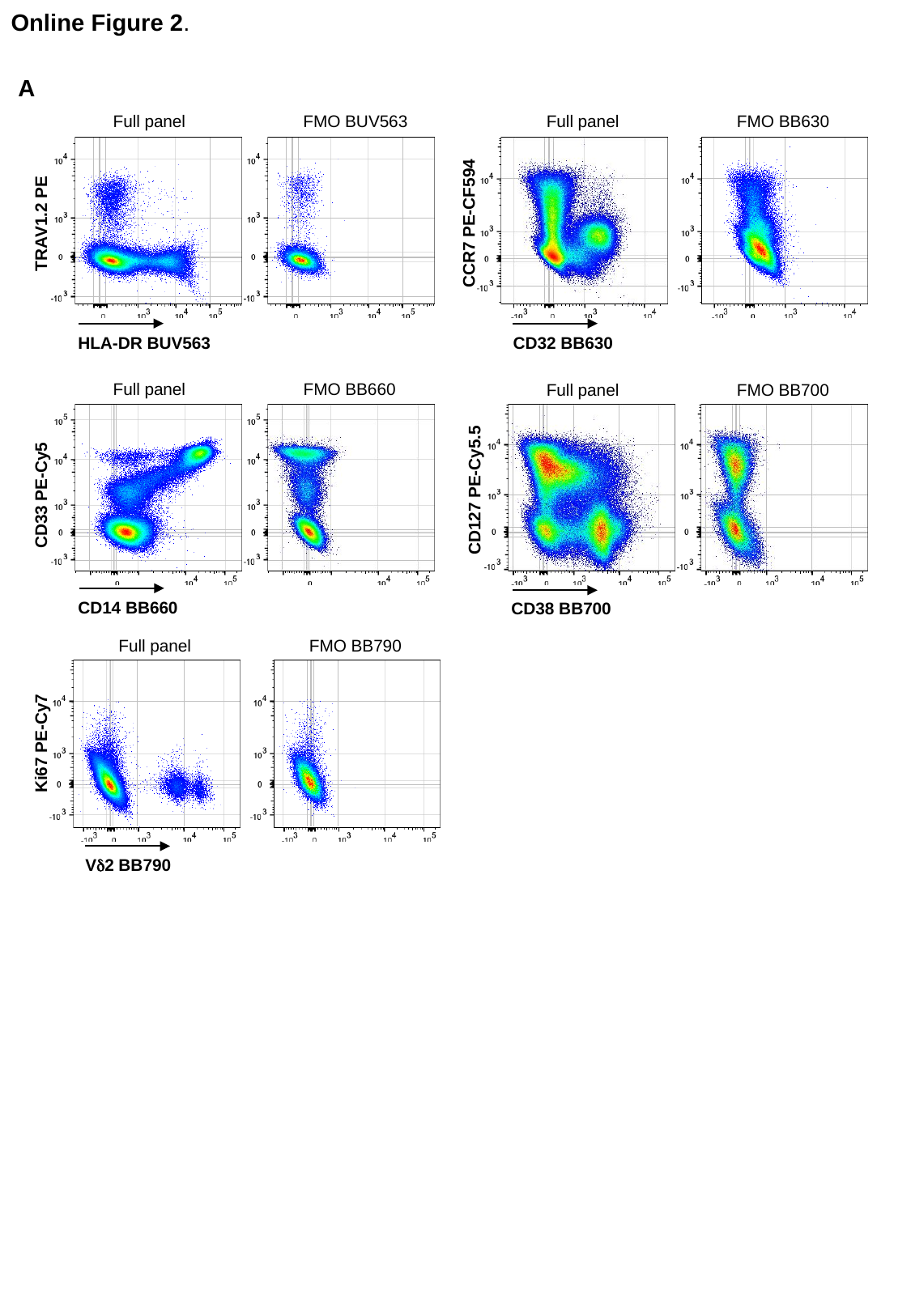

Online Figure 2.
A
Full panel
FMO BUV563
Full panel
FMO BB630
TRAV1.2 PE
CCR7 PE-CF594
CD32 BB630
HLA-DR BUV563
Full panel
FMO BB660
Full panel
FMO BB700
CD127 PE-Cy5.5
CD33 PE-Cy5
CD14 BB660
CD38 BB700
Full panel
FMO BB790
Ki67 PE-Cy7
Vd2 BB790

## Slide 3
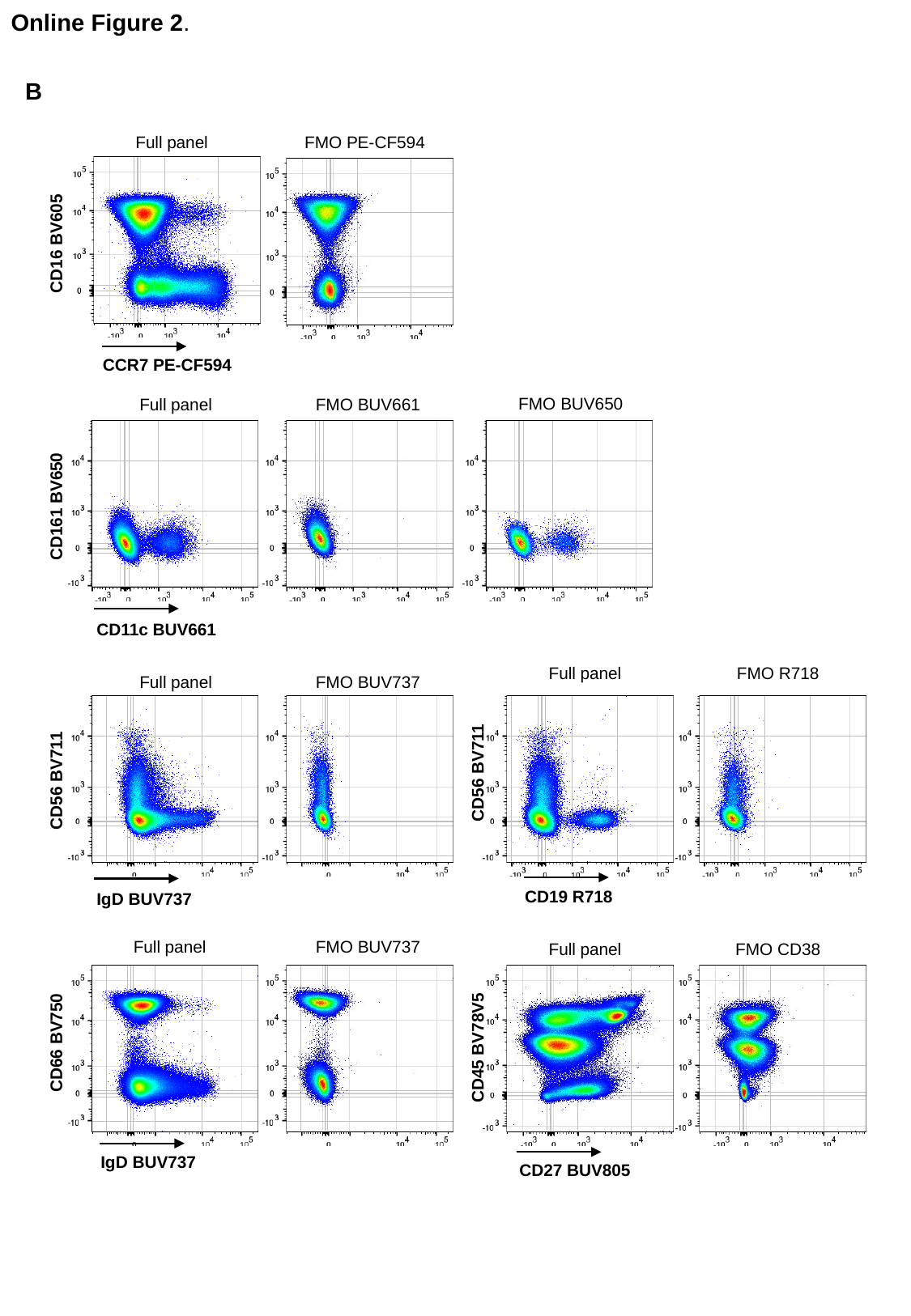

Online Figure 2.
B
Full panel
FMO PE-CF594
CD16 BV605
CCR7 PE-CF594
FMO BUV650
Full panel
FMO BUV661
4.33
CD161 BV650
CD11c BUV661
Full panel
FMO R718
Full panel
FMO BUV737
CD56 BV711
CD56 BV711
CD19 R718
IgD BUV737
Full panel
FMO BUV737
Full panel
FMO CD38
CD66 BV750
CD45 BV78V5
IgD BUV737
CD27 BUV805

## Slide 4
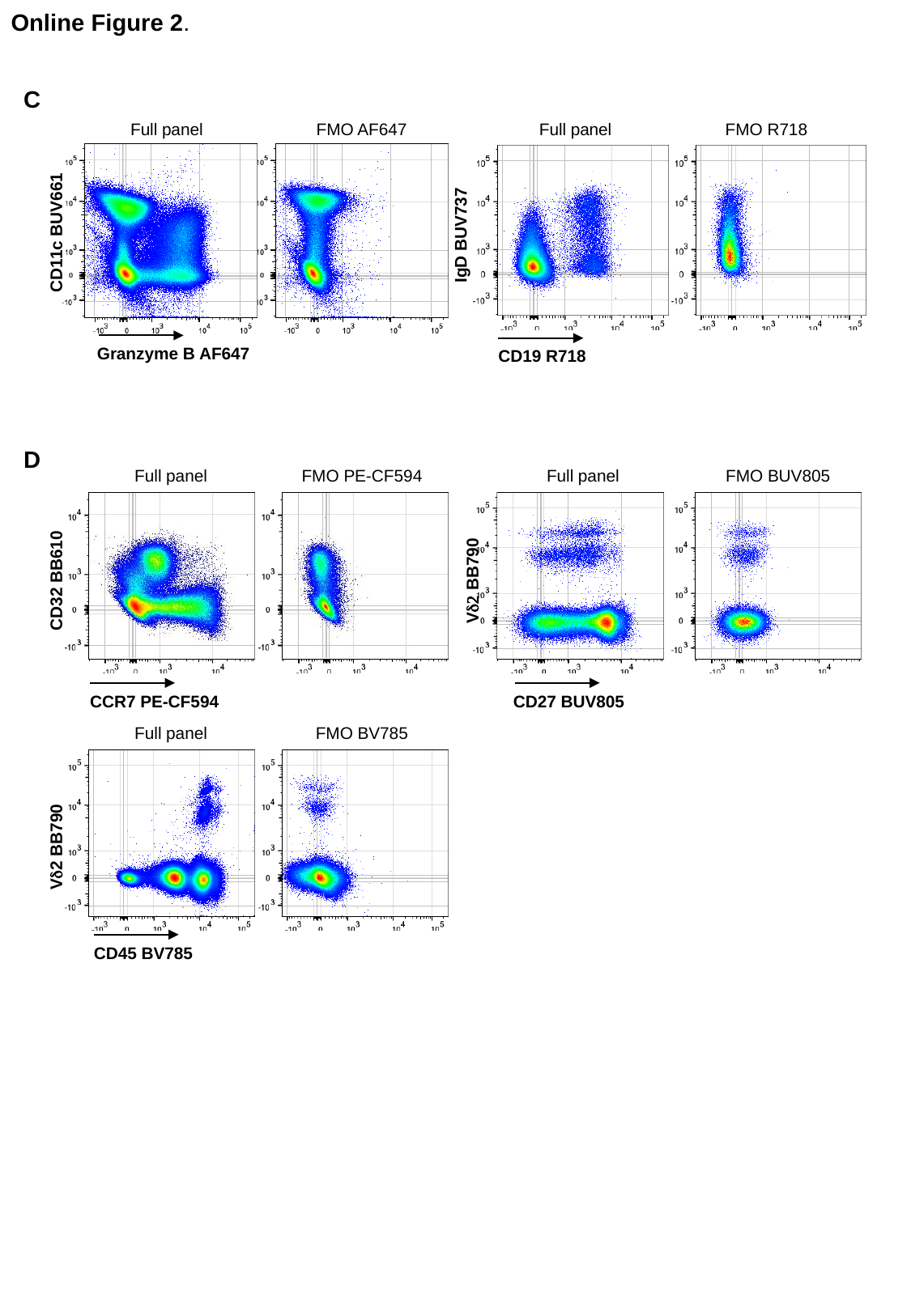

Online Figure 2.
C
Full panel
FMO AF647
Full panel
FMO R718
CD11c BUV661
IgD BUV737
Granzyme B AF647
CD19 R718
D
Full panel
FMO PE-CF594
Full panel
FMO BUV805
CD32 BB610
Vd2 BB790
CCR7 PE-CF594
CD27 BUV805
Full panel
FMO BV785
Vd2 BB790
CD45 BV785

## Slide 5
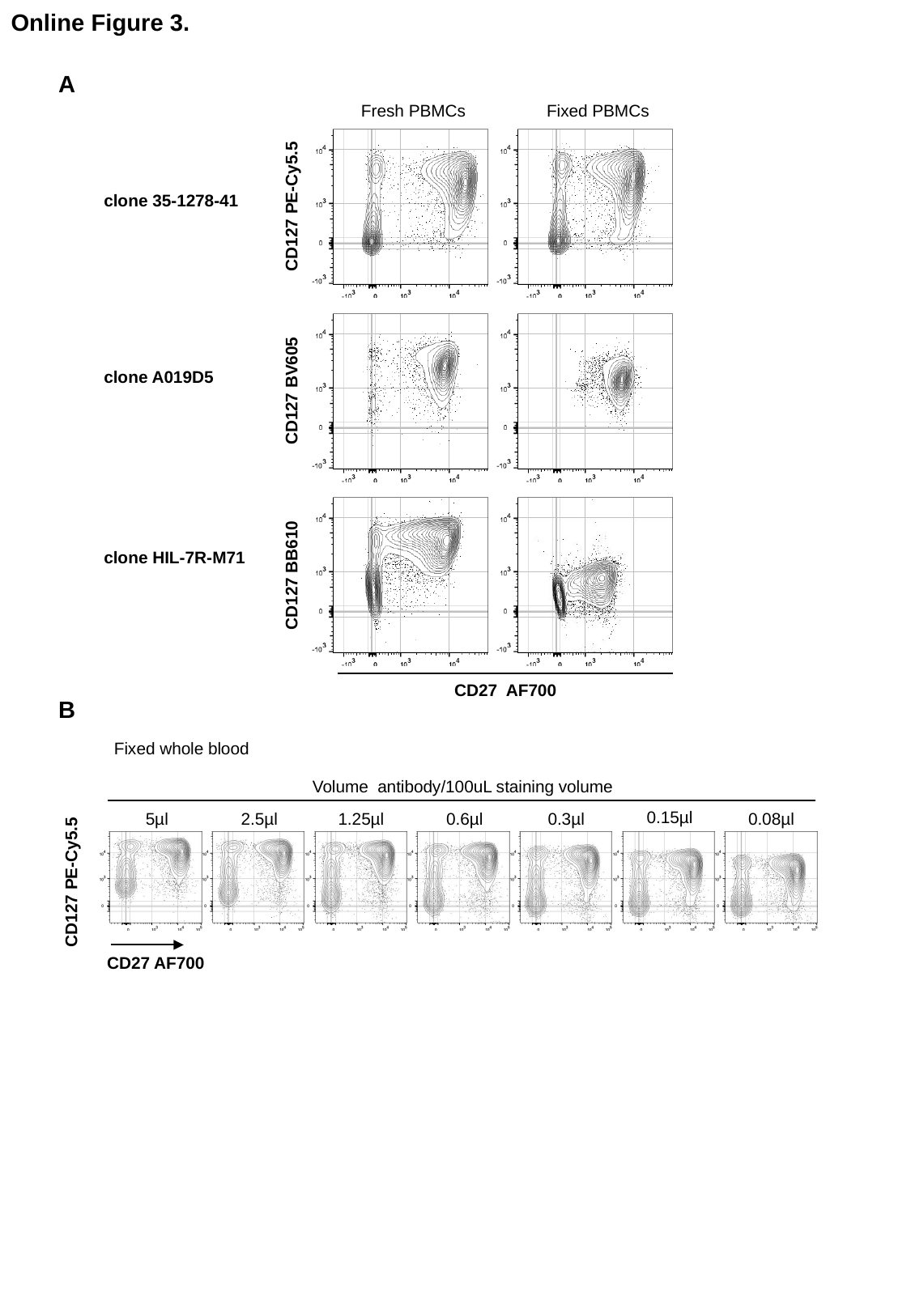

Online Figure 3.
A
Fresh PBMCs
Fixed PBMCs
clone 35-1278-41
CD127 PE-Cy5.5
clone A019D5
CD127 BV605
clone HIL-7R-M71
CD127 BB610
CD27 AF700
B
Fixed whole blood
Volume antibody/100uL staining volume
0.15µl
0.3µl
0.08µl
0.6µl
1.25µl
5µl
2.5µl
CD127 PE-Cy5.5
CD27 AF700

## Slide 6
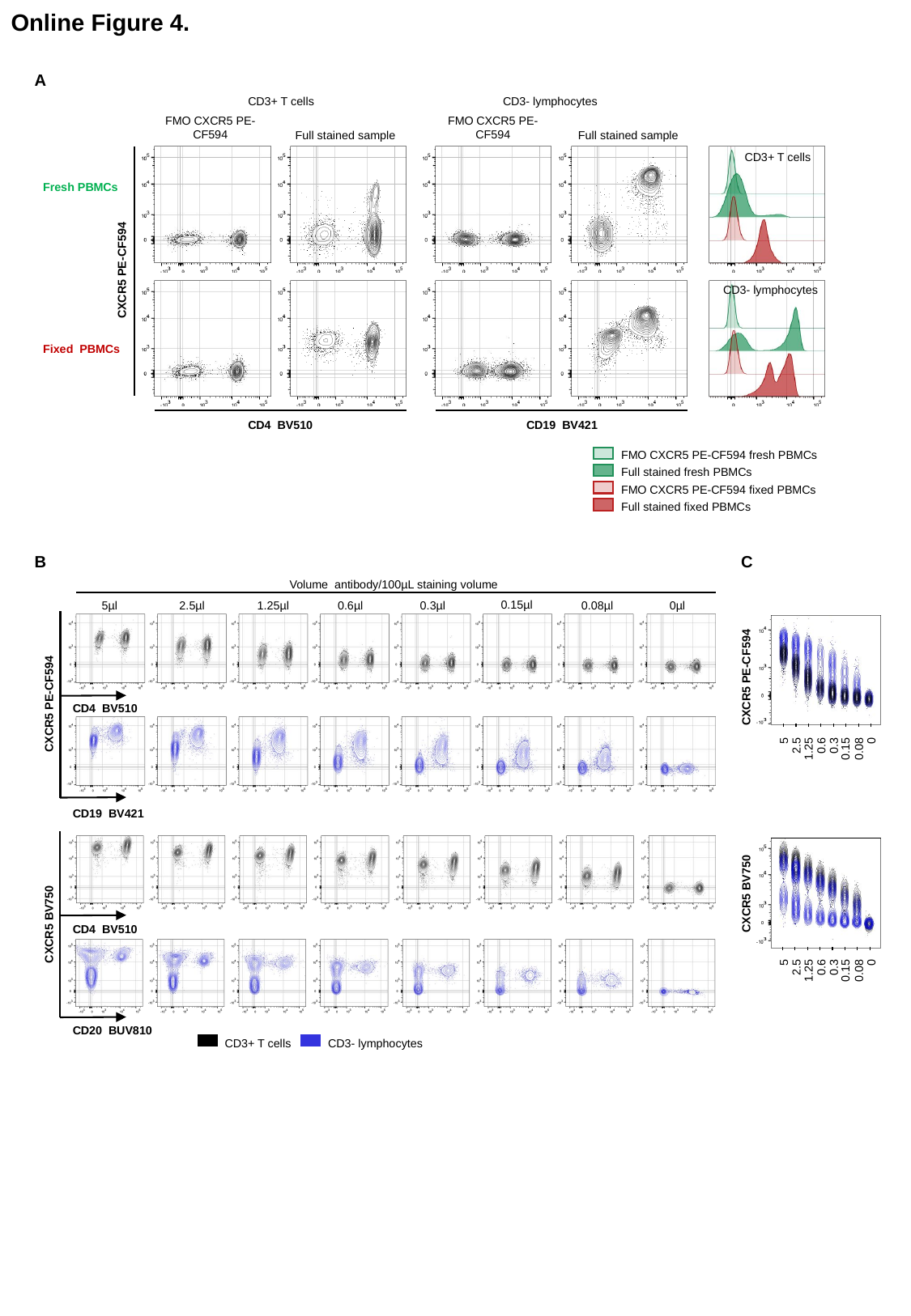

Online Figure 4.
A
CD3+ T cells
CD3- lymphocytes
FMO CXCR5 PE-CF594
FMO CXCR5 PE-CF594
Full stained sample
Full stained sample
CD3+ T cells
Fresh PBMCs
CXCR5 PE-CF594
CD3- lymphocytes
Fixed PBMCs
CD4 BV510
CD19 BV421
FMO CXCR5 PE-CF594 fresh PBMCs
Full stained fresh PBMCs
FMO CXCR5 PE-CF594 fixed PBMCs
Full stained fixed PBMCs
B
C
Volume antibody/100µL staining volume
0.15µl
0.3µl
0.08µl
0µl
1.25µl
0.6µl
5µl
2.5µl
CXCR5 PE-CF594
CXCR5 PE-CF594
CD4 BV510
0
5
2.5
0.6
0.15
0.08
1.25
0.3
CD19 BV421
CXCR5 BV750
CXCR5 BV750
CD4 BV510
0
5
2.5
0.6
0.15
0.08
1.25
0.3
CD20 BUV810
CD3+ T cells
CD3- lymphocytes

## Slide 7
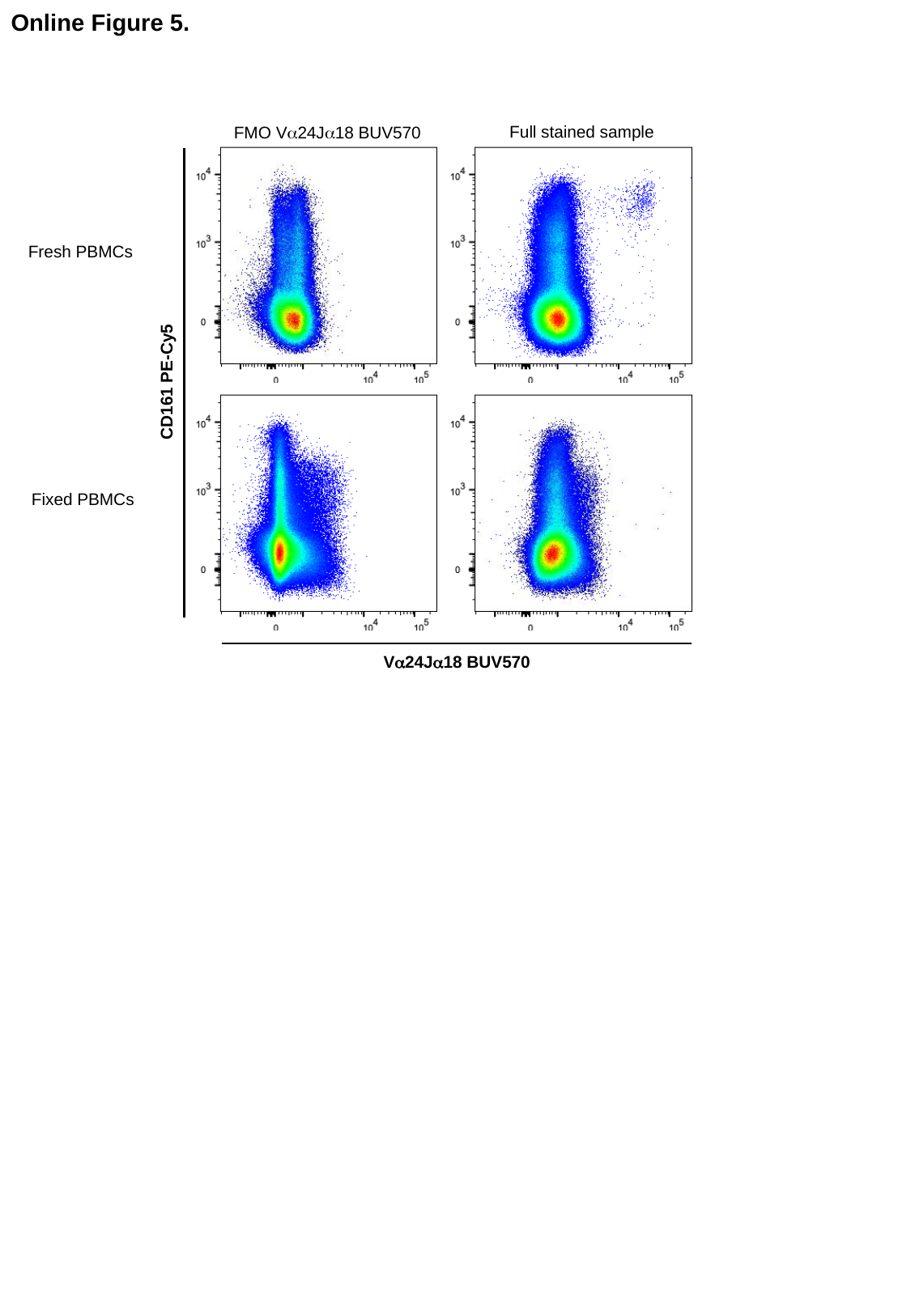

Online Figure 5.
Full stained sample
FMO Va24Ja18 BUV570
Fresh PBMCs
CD161 PE-Cy5
Fixed PBMCs
Va24Ja18 BUV570

## Slide 8
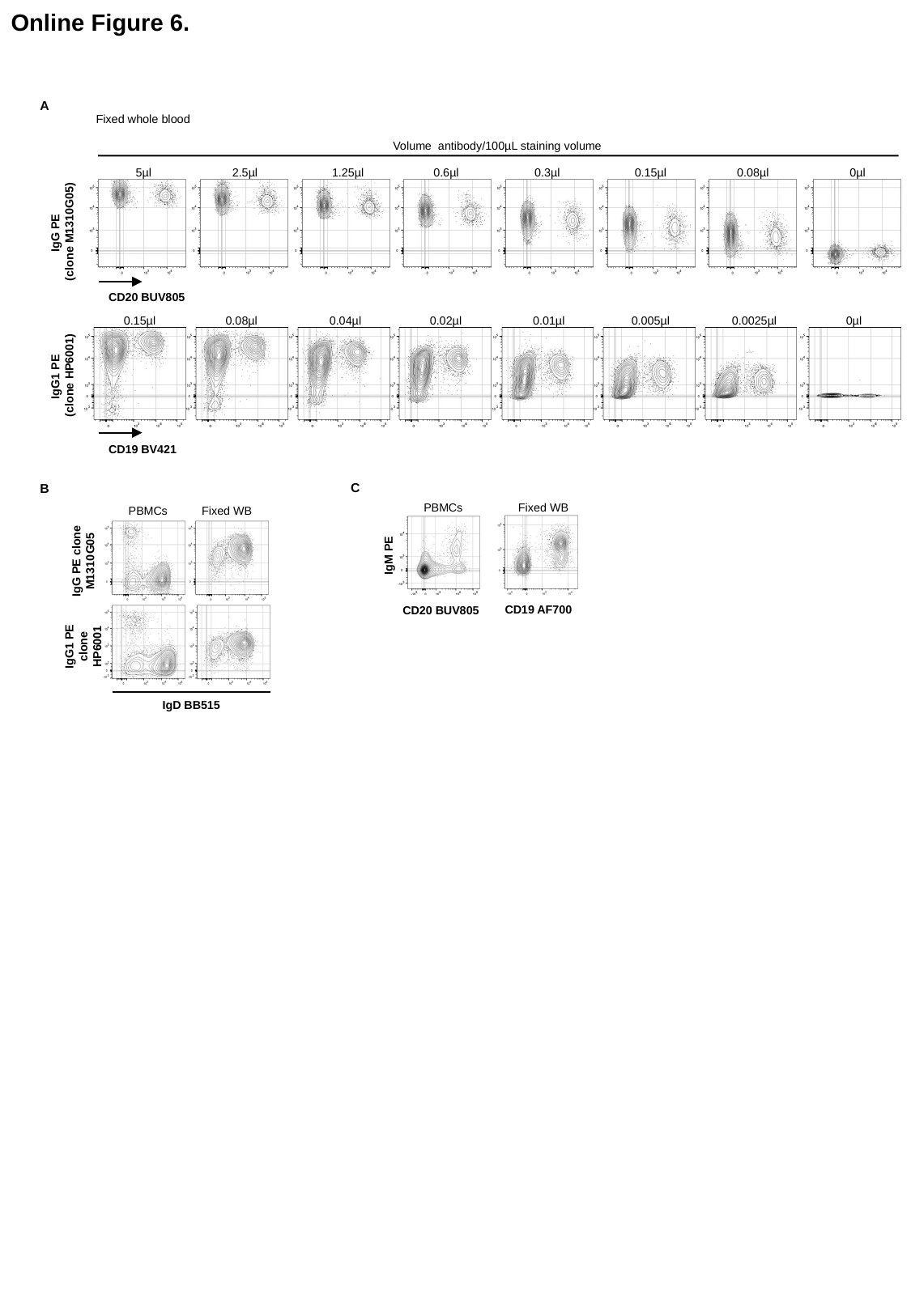

Online Figure 6.
A
Fixed whole blood
Volume antibody/100µL staining volume
5µl
2.5µl
1.25µl
0.6µl
0.3µl
0.15µl
0.08µl
0µl
IgG PE
(clone M1310G05)
CD20 BUV805
0.15µl
0.08µl
0.04µl
0.02µl
0.01µl
0.005µl
0.0025µl
0µl
IgG1 PE
(clone HP6001)
CD19 BV421
C
B
PBMCs
Fixed WB
PBMCs
Fixed WB
IgG PE clone M1310G05
IgM PE
CD19 AF700
CD20 BUV805
IgG1 PE clone HP6001
IgD BB515

## Slide 9
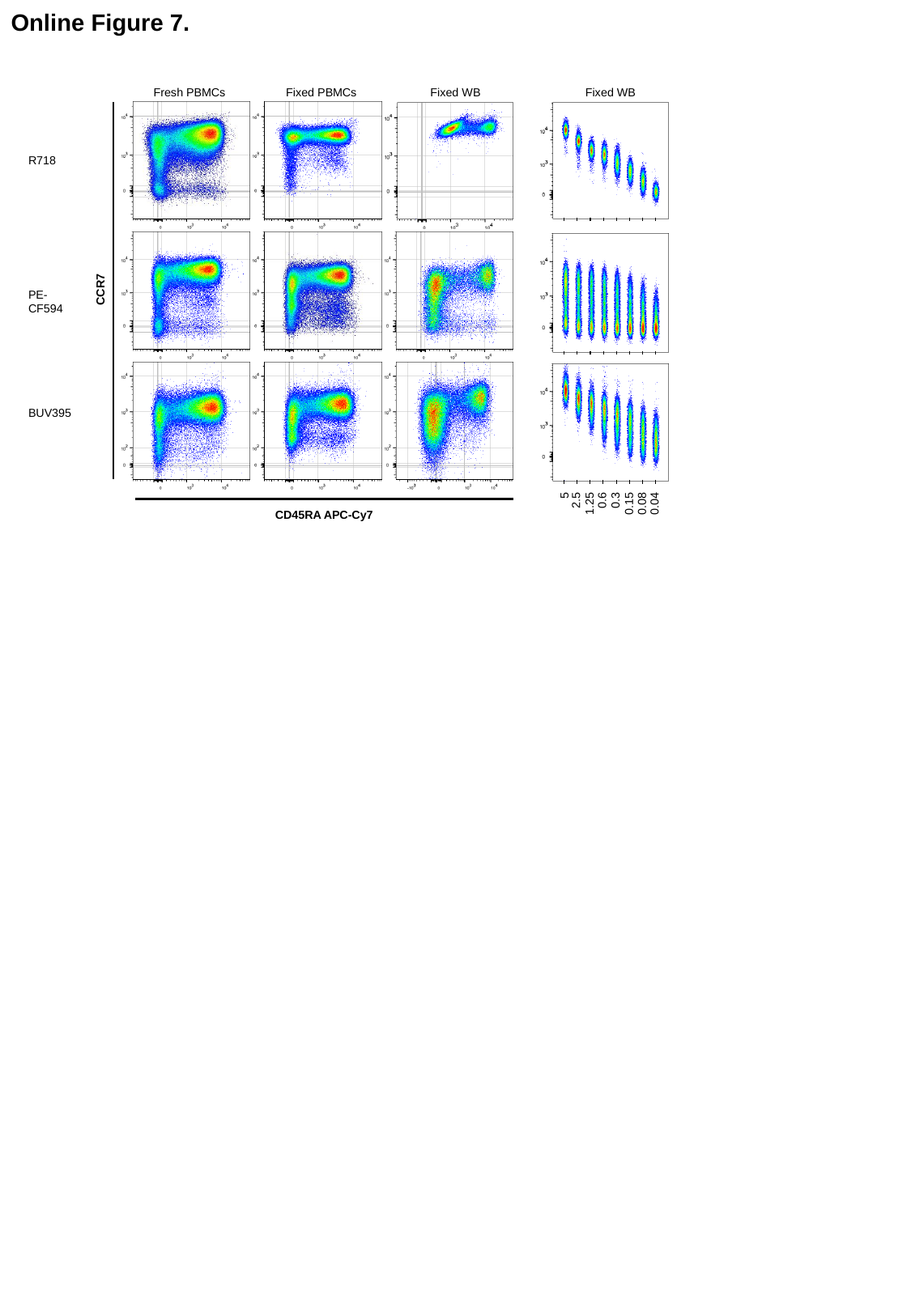

Online Figure 7.
Fresh PBMCs
Fixed PBMCs
Fixed WB
Fixed WB
R718
CCR7
PE-CF594
BUV395
5
0.6
2.5
0.15
0.3
0.04
1.25
0.08
CD45RA APC-Cy7

## Slide 10
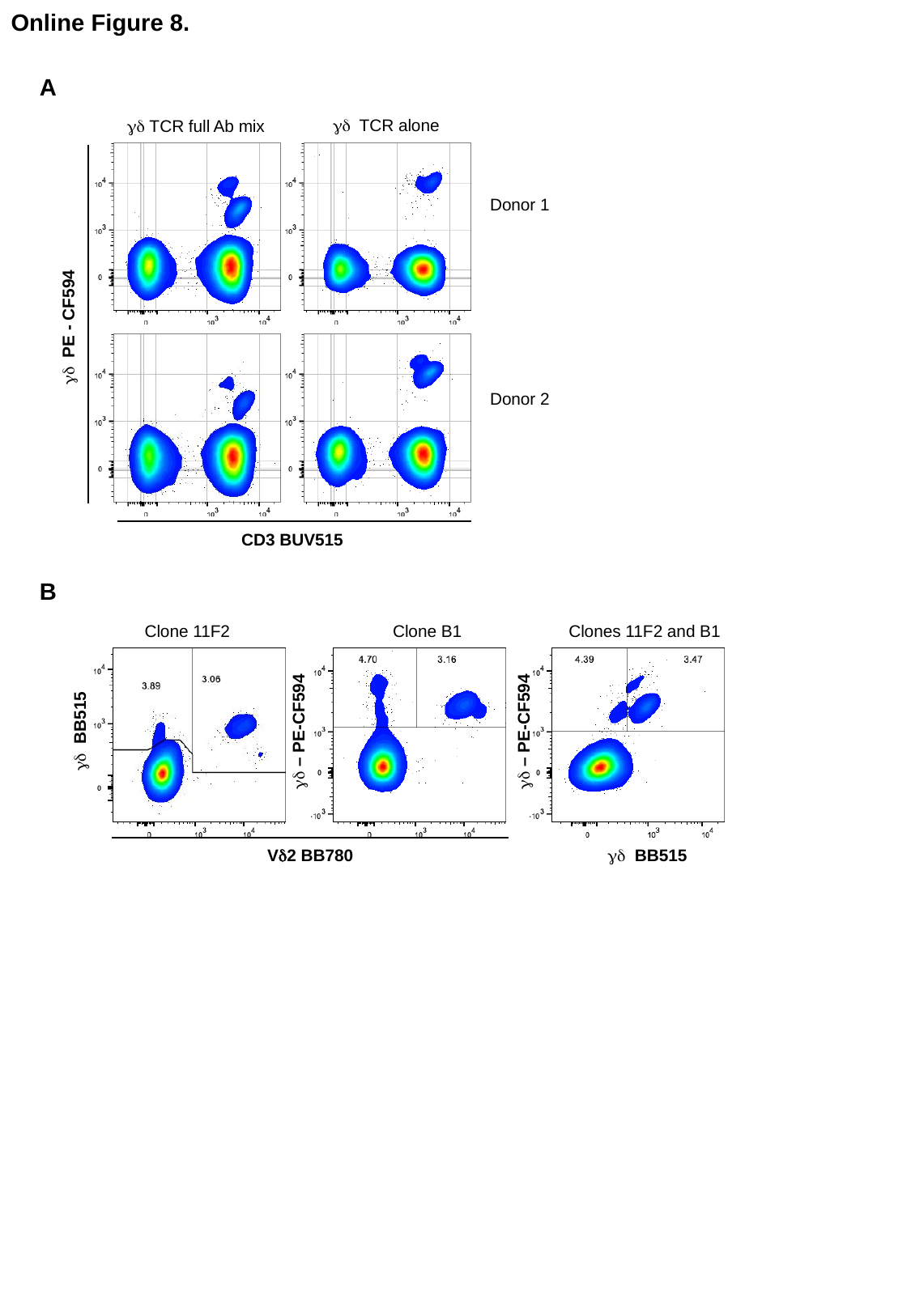

Online Figure 8.
A
gd TCR alone
gd TCR full Ab mix
Donor 1
gd PE - CF594
Donor 2
CD3 BUV515
B
Clone 11F2
Clone B1
Clones 11F2 and B1
gd BB515
gd – PE-CF594
gd – PE-CF594
Vd2 BB780
gd BB515

## Slide 11
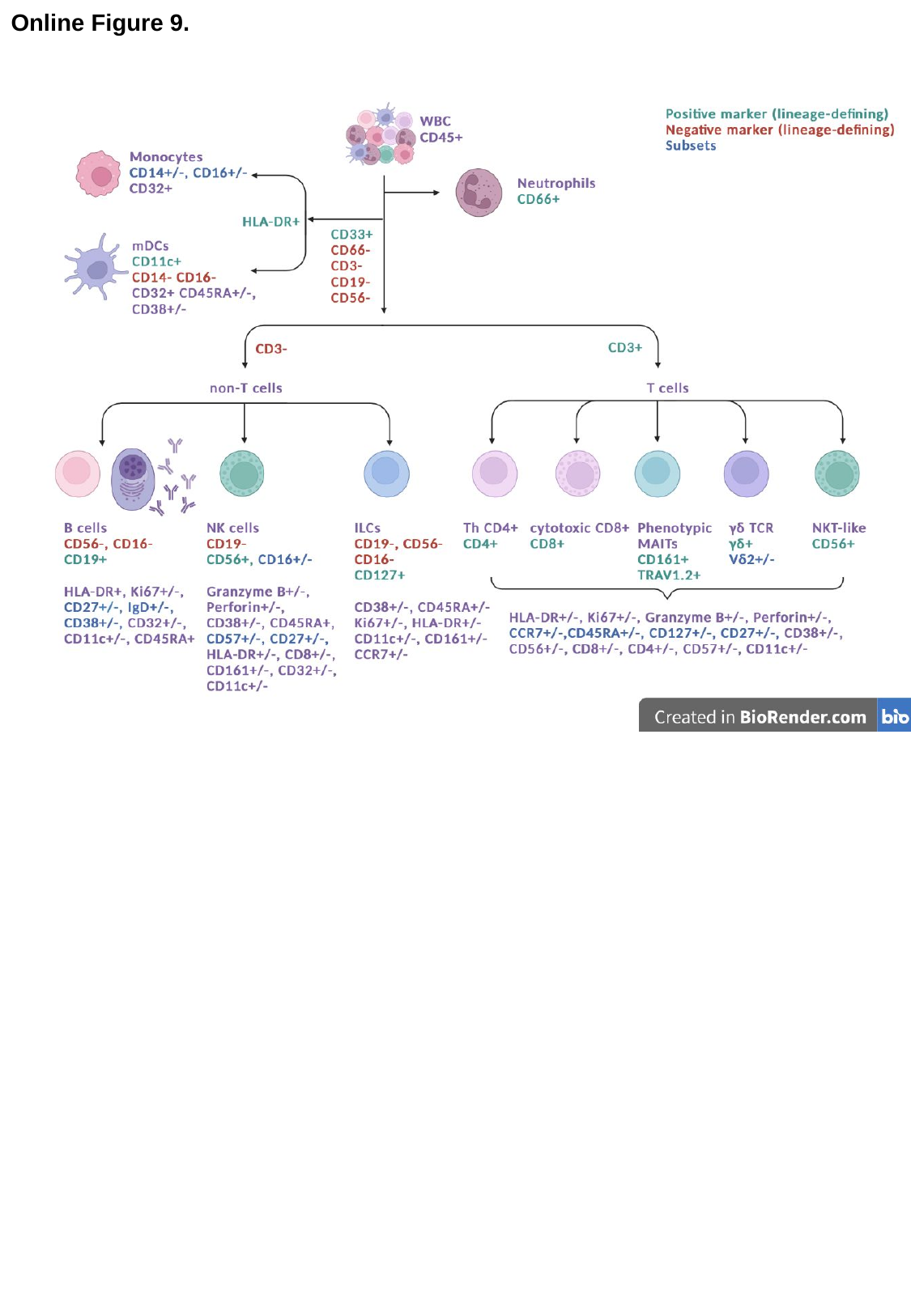

Online Figure 9.
