# Supplementary material for: OMIP-101: 27-color flow cytometry panel for immunophenotyping of major leukocyte populations in fixed whole blood
Source: Cytometry A. Author manuscript; Available in PMC 2024 Mar 22. (PMC10958279; doi:10.1002/cyto.a.24827)
Supplement: Supinfo2 [file NIHMS1964512-supplement-Supinfo2.docx]

**Online Material**

**OMIP-0XX: 27-color flow cytometry panel for immunophenotyping of major leukocyte populations in cryopreserved human fixed whole blood**

Claire Imbratta^1^, Tim Reid^1^, Asma Toefy^1^, Thomas J. Scriba^1^ and Elisa Nemes^1^

^1^South African Tuberculosis Vaccine Initiative, Division of Immunology, Department of Pathology, Institute of Infectious Disease and Molecular Medicine, University of Cape Town, Cape Town, South Africa

**Antibody panel development strategy**

We developed this 27-color antibody panel using two Becton Dickinson (BD) FACSymphony A5 instruments and a BD Fortessa. Instrument configurations are shown in **Online Table 1**. While BD FACSymphony A5 cytometer A was mainly used for testing antibody clones on fixed whole blood (WB), we designed this panel and performed most iterations on BD FACSymphony A5 cytometer B. BD FACSymphony A5 cytometer A and B differ in one of the laser wavelengths (green 532nm vs yellow-green 561nm) and power (red, blue and UV lasers). Additionally, most of the filters were slightly different (detectors R710, R660, G660, G575, B780, B710, B660, B515, V780, V655, V610, V570, V510, V450, U780, U730, U660, U570, U500, U395). BD FACSymphony A5 cytometer B has additional detectors (B750, U600) compared to cytometer A, which were not used for this panel. The BD Fortessa cytometer C was used for preliminary testing of staining performance of antibodies conjugated with the fluorochromes compatible with this instrument. All details about markers included in the final version of this panel are shown in **Online Table 2**. This panel was initially developed to study potential immune correlates of protection using fixed WB samples collected from participants of clinical trials of tuberculosis (TB) vaccines. We therefore prioritized markers based on their biological relevance to TB immunity (**Online Table 3**). Markers used to define major leukocyte lineages including granulocytes, monocytes, dendritic cells (DCs), T cells, B cells and natural killer (NK) cells were given highest priority. Since phenotypic markers associated with cell subset differentiation and activation depend on expression of lineage markers, they were assigned lower priority. Cytolytic markers were given even lower priority due to their expression on certain cell subsets, including NK and T cells. CD11c and CD32 were assigned least priority due to their assumed modest importance in TB immunity.

Following priority assignment of selected markers, we continued designing our panel by pairing each antibody to a fluorochrome using spill-over spreading matrix (SSM; **Online Table 4**). While low-abundance markers (expressed at low levels) were assigned to bright dyes with least total spread received, abundant markers (expressed at high levels) were paired with dim dyes. Importantly, fluorochromes with spread into multiple other channels were paired with markers that are not co-expressed on the same cell type, and taking into account hierarchical gating that would exclude the “high-spread” cell subset prior to analysing the cell subset receiving the spread.

All antibodies were titrated using at least 8 concentrations in a two-fold dilution series on fixed WB. Although reagents were titrated on both instruments, we showed titers selected on flow BD FACSymphony A5 cytometer B in **Online Figure 1**. Multiple reagents were titrated simultaneously, along with co-staining with a constant amount of “anchor markers”, when required. **Online Table 5** provides details of staining combinations for all titrations, along with the parent gate used to generate **Online Figure 1**. For each reagent, optimal concentration was selected based on stain index, spreading magnitude into other channels and consistent detection of expression. The following formula was used to calculate the stain index:

Stain Index = [MFI positive population – MFI negative population] / [2* rSD negative population] where MFI is Median Fluorescent Intensity and rSD robust Standard Deviation. All reagents were titrated on blood from healthy donors and gated on the most relevant leukocyte populations (e.g NK cells for granzyme B), except for CD45 and CD66, which were gated on singlets (**Online Table 5)**. Importantly, markers that are known to be expressed at different levels by multiple cell subsets (e.g HLA-DR, CD16), were assessed on all relevant subtypes.

The impact of spread and cross-laser excitation was assessed using the SSM in combination with fluorescence minus-one-controls (FMO, **Online Figure 2** and **Online Table 4**). Along with those excited by the violet laser, fluorochromes excited by the yellow-green laser contribute to the highest spread into other channels. We noticed substantial cross-laser excitation of the fluorochromes included in this panel (mostly tandem dyes) particularly between yellow-green and blue as well as between violet and UV lasers. Minor manual adjustments of the compensation matrix were required. These were consistent across experiments and predictable based on cross-laser excitation. These adjustments were made based on visualization of NbyN plots (i.e. every marker against every other marker), and the expected staining patterns based on the FMO experiment. Since all CD14+ monocytes express CD33, we decided to pair them to BB660 and PE-Cy5, respectively, two fluorochromes with high spread. Given that proliferating γδ T cells are known to be particularly rare in WB, we assigned two bright fluorochromes PE-Cy7 and BB790, to Ki67 and Vδ2 (exclusively expressed by γδ T cells), respectively. Although naïve and central memory T cells, partially expressing CD127, can upregulate CD38 following activation, the fluorochrome combination of CD127 with PE-Cy5.5 and CD38 with BB700 provides reasonable resolution (**Online Figure 2A**). Fluorochromes tested in this assay that are excited by the red laser showed minimal spread into other channels. Anti-CD161 conjugated with BV650, mainly used to identify phenotypic mucosa-associated invariant T (MAIT) cells in this panel, is a particularly dim antibody-fluorochrome combination. Therefore, spread into U660 detector is minor (**Online Figure 2B)**. The BV711-conjugated antibody for the lineage marker of NK and NKT-like cells, CD56, contributes spread into U730 and R710 detectors. We decided to assign antibodies for the B cell markers IgD to BUV737 (measured by U730 detector) and CD19 to R718 (measured by R710 detector). To avoid poor resolution induced by spread from BV750 into U730 detector, we paired BV750 to anti-CD66 to exclusively identify granulocytes, which are gated out early in the hierarchical gating strategy (**Online Figure 2B)**. We chose to assign anti-CD45 to BV785 since all CD27+ cells (labelled with BUV805, receiving spread from BV785 are leukocytes (**Online Figure 2B)**. Anti-CD11c BUV661, which stains various cell subsets including myeloid cells at high level, induces substantial spread into R660 detector, used to measure AF647. To allow satisfactory resolution, we allocated AF647 to the anti-granzyme B antibody, used to define cytotoxic lymphocytes, which do not co-express CD11c at high levels (**Online Figure 2C**).

Laser cross-excitation was observed to a lesser extent between blue and violet lasers. Although partially co-expressed, Vδ2 BB790 and CD27 conjugated to BUV805, also known as a bright fluorochrome, allow good resolution in this panel. Spread from Vδ2 BB790 into V780 detector (used to measure CD45 BV785) does not appear problematic due to expression of CD45 by all γδ T cells (**Online Figure 2D**).

The list of reagents tested, with reasons for exclusion from the final panel, is described in **Online Table 6**. Importantly, this panel shares most markers with another published 27-color panel developed for fresh and fixed WB (1). Additional iterations were performed to allow better characterization of lineage populations and their phenotype. Anti-CD11b labelled with BV750, initially included to identify myeloid-derived suppressor cells (MDSCs), was replaced by anti-CD66 to improve identification of granulocytes and exclusion of this highly autofluorescent cell subset. We tested CD11b-BUV615, but decided to remove it from this panel due to high spread into G610 detector (used to measure CCR7 PE-CF594) and extremely low frequencies of MDSCs in WB. In addition, anti-CD20 was replaced by anti-CD19 to allow better identification of the rare plasmablast subset. As described thereafter, anti-γδ TCR and anti-CCR7, initially paired with BUV395 and PE-CF594, respectively, were interchanged to allow better resolution of CCR7 on T cells. Finally, due to repeated tandem dissociation of APC-Cy7 before the reagent's expiry date, CD45RA-APC-Cy7 was replaced by APC-Efluor780.

A recurrent reason for non-inclusion of certain antibody conjugates was poor staining, leading to suboptimal separation of negative and positive cells, following fixation of WB and/or peripheral blood mononuclear cells (PBMCs). Thawed PBMCs were stained either before (fresh) or after fixation in FACS Lysing solution (BD Biosciences) following the same protocol as the DLC-ICE samples. In fact, as fixative can substantially affect staining or detection of certain epitopes, appropriate staining with adequate clones and conjugates was carefully investigated. For example, staining of PBMCs with different anti-CD127 clone/conjugate combinations revealed that only clone 35-1278-41 yielded appropriate staining of fixed cells (**Online Figure 3A**). Titration on fixed WB is shown in **Online Figure 3B**.

Similarly, we noticed that fixative affected staining of CXCR5 (clone J232D4), expressed by T follicular helper (Tfh) and B cells, resulting in poor separation compared to fresh (not fixed) PBMCs (**Online Figure 4A**). Surprisingly, anti-CXCR5 conjugates (PE-CF594 and BV750) stain B cells from fixed WB adequately, while no signal was detected on CD4 T cells (**Online Figure 4B-C)**. Therefore, this marker and PD-1, also used to define T follicular helper cells, were not included in the panel.

After testing of MR1-5-OP-RU and CD1d-αGalCer tetramers for inclusion in this panel, we noted non-specific staining on CD3- T cells and poor separation of stained cells, respectively (not shown). This justified replacement of these tetramers with antibodies for phenotypic markers for MAIT cells, TRAV1.2 and CD161. Similarly, we could not include anti-Vα24Jα18, which detects iNKT cells which express these TCR chains, due to insufficient signal observed following PBMC fixation (**Online Figure 5**). We therefore compromised to identify “NKT-like” as CD56+ T cells rather than the more accurate definition based on CD1d-αGalCer tetramer or invariant TCR staining.

Since increasing evidence suggests that B cells and antibodies may play a role in TB immunity, we tested several anti-immunoglobulin isotype antibodies to include in this panel. We found that fixation affected anti-IgG staining with 2 different clones (M1310G05 and HP6001, **Online Figure 6A and C**), but not anti-IgD and anti-IgM staining (**Online Figure 6B and C**). We therefore did not include anti-IgG in the panel.

We also noticed that the same antibody clone conjugated with different fluorochromes reacted differently to fixative. For example, we compared staining with one anti-CCR7 clone (150503) labelled with 3 different fluorochromes (R718, PE-CF594 and BUV395) before and after fixation. While resolution between fluorochromes appeared similar on fresh PBMCs, we observed substantially different patterns on fixed PBMCs and WB. Differences were most noticeable between PE-CF594/BUV395 and R718. In fact, anti-CCR7 labelled with R718 resulted in no resolution on fixed cells (**Online Figure 7**). Anti-CCR7 was stained separately, before the main antibody cocktail was added, because optimal staining requires incubation at 37°C (not shown).

Interestingly, we noticed increased resolution when anti-γδ TCR reagent was stained separately from the antibody cocktail, as previously shown by Park et al (2) (**Online Figure 8A**). This improvement may be triggered by the absence of binding competition between anti-Vδ2, also present in this panel, and anti-γδ TCR. When comparing 11F2 and B1 clones of the anti-γδ TCR antibody, we observed that the B1 clone may improve capture of Vδ2-negative γδ T cells, while γδ T cells co-expressing Vδ2 may not be affected by the clone selection (**Online Figure 8B**).

We noticed that the resolution of anti-CD27 staining decreased when it was stained with the full panel, compared to the titrations. Staining anti-CD27 separately at 37°C, before the main antibody cocktail was added, seemed to slightly improve resolution (not shown). This was not a critical difference, but since we already had a separate staining step for anti-CCR7 and anti- γδ TCR, we also included anti-CD27 in this small cocktail and stained separately.

As described in the hierarchical gating strategy from **Figure 1**, we were able to identify all major leukocyte subsets and assess expression of multiple phenotypic markers on each lineage cell population. A schematic illustrating marker expression on each population is shown in **Online Figure 9.**

Finally, we also tested pre-incubating the samples with Fc block, but no noticeable improvement was observed (not shown). We therefore did not include a Fc blocking step in our staining protocol.

**Materials and Reagents**

- PBS (Lonza BioWhittaker, cat # BE17-517Q)
- Perm/Wash (BD, cat # 554723)
- Brilliant Stain Buffer (BD, cat # 566349)
- References beads: Precision count beads (BioLegend, cat # 424902) or

Flow-Count Fluorospheres (Beckman Coulter, cat # 7547053)

- Compensation beads (BD, cat # 552843)
- Antibodies listed in **Online Table 2**.
- Cryopreserved DLC-ICE samples as described in (3); different donors were used throughout the panel optimization.

**Staining procedure**

1. Thaw cryopreserved DLC-ICE cell vials (containing cells from 200 μl of WB) gently in a water bath pre-warmed at 37°C. Wipe the sides of the cryovial carefully with an alcohol swab to avoid contamination with water from the water-bath.
2. Transfer the thawed cell suspension into its respective 5mL tube. Rinse the vial with 1mL of 1X PBS and add into the tube.
3. Centrifuge the tubes at 2100 rcf for 7 min at room temperature.
4. As soon as the centrifuge has stopped, check for cell pellets and decant the supernatant in a single motion. Blot the remaining supernatant onto a paper towel.
5. Add 1mL of 1X Perm/Wash solution to each tube and pulse vortex 10 times. Check for presence of cell clumps and disrupt them with a p1000 if present. Incubate for 10 min at room temperature in the dark.
6. Centrifuge as described in 3 above.
7. Stain cells with anti-CCR7, anti-CD27 and anti-γδ TCR antibodies at optimal concentration diluted in 25 μL 1X Brilliant Stain Buffer (for a total staining volume of ~50 μL) and incubate for 30 min at 37°C in the dark.
8. While incubating, prepare the antibody cocktail for staining, diluting them in 50µL of 50:50 Brilliant Stain Buffer: 1X Perm/Wash. Centrifuge antibody cocktail at 10 000 rcf for 5 min to remove aggregates.
9. Without washing, add 50 μL of antibody cocktail to the cells (for a final volume ~100 μL).
10. Incubate 30 min at 4°C in the dark.
11. After incubation, add 2 mL of 1X Perm/Wash to each tube, vortex and centrifuge at 2100 rcf for 7 min at room temperature.
12. Decant the supernatant in a single motion and blot the remaining supernatant on paper towel. Vortex to resuspend the cell pellet.
13. Add 50 μL PBS 0.5-1% PFA to each tube.
14. Thoroughly vortex the reference beads, use reverse pipetting technique, wipe the external part of the tip with clean absorbent paper and add 100 μL of reference beads to each tube. Keep on ice in the dark until acquisition on the flow cytometer. To improve accuracy of absolute count results, we recommend to acquire at least 20 000 reference beads per sample.

**Online Table 1. Instrument configurations**

**BD FACSymphony A5 cytometer A**

| **Laser**  **Wavelength (nm)** | **Laser Power (mW)** | **Laser Type** | **Detector** | **Dichroic LP Filter (nm)** | **Band Pass (nm)** | | **Fluorochrome** | | |  |
| --- | --- | --- | --- | --- | --- | --- | --- | --- | --- | --- |
| 637  (Red) | 200 | DPSS | R780 | 750 | 780/60 | | APC-eFluor780 | | |  |
|  |  |  | R710 | 685 | 730/45 | | R718 | | |  |
|  |  |  | R660 | 630 | None | | AF647 | | |  |
| 532  (Green) | 200 | DPSS | G780 | 750 | 780/60 | | PE-Cy7 | | |  |
|  |  |  | G710 | 690 | 710/50 | | PE-Cy5.5 | | |  |
|  |  |  | G660 | 635 | 660/40 | | PE-Cy5 | | |  |
|  |  |  | G610 | 600 | 610/20 | | PE-CF594 | | |  |
|  |  |  | G575 | - | 575/25 | | PE | | |  |
| 488  (Blue) | 100 | DPSS | B780 | 740 | 780/40 | | BB790 | | |  |
|  |  |  | B710 | 690 | 710/50 | | BB700 | | |  |
|  |  |  | B660 | 635 | 660/40 | | BB660 | | |  |
|  |  |  | B610 | 600 | 610/20 | | BB630 | | |  |
|  |  |  | B515 | 505 | 515/20 | | BB515 | | |  |
|  |  |  | SSC | - | 488/10 | |  | | |  |
| 405  (Violet) | 200 | DPSS | V780 | 770 | 785/62 | | BV785 | | |  |
|  |  |  | V750 | 735 | 750/30 | | BV750 | | |  |
|  |  |  | V710 | 685 | 710/40 | | BV711 | | |  |
|  |  |  | V655 | 630 | 660/20 | | BV650 | | |  |
|  |  |  | V610 | 580 | 605/20 | | BV605 | | |  |
|  |  |  | V570 | 550 | 575/25 | | BV570 | | |  |
|  |  |  | V510 | 505 | 510/20 | | BV510 | | |  |
|  |  |  | V450 | - | 450/40 | | BV450 | | |  |
| 355 (Ultraviolet) | 65 | DPSS | U780 | 755 | 785/62 | | BUV805 | | |  |
|  |  |  | U730 | 710 | 740/35 | | BUV737 | | |  |
|  |  |  | U660 | 635 | 660/40 | | BUV661 | | |  |
|  |  |  | U570 | 550 | 570/40 | | BUV563 | | |  |
|  |  |  | U500 | 480 | 515/30 | | BUV496 | | |  |
|  |  |  | U450 | 410 | 450/50 | |  | | |  |
|  |  |  | U395 | - | 379/28 | | BUV395 | | |  |
|  |  |  |  |  | |  | |  |  | |
|  |  |  |  |  | |  | |  |  | |

Abbreviations: LP = Long Pass; DPSS = diode-pumped solid-state laser; APC= Allophycocyanin; AF = Alexa Flour; PE= Phycoerythrin; Cy= Cyanin; CF= Cyanin-based fluorescent dyes; BV= Brilliant Violet, BUV= Brilliant Ultraviolet; BB= Brilliant Blue.

**BD FACSymphony A5 cytometer B**

| **Laser**  **Wavelength (nm)** | **Laser Power (mW)** | **Laser Type** | **Detector** | | **Dichroic LP Filter (nm)** | | **Band Pass (nm)** | | **Fluorochrome** | | |  |
| --- | --- | --- | --- | --- | --- | --- | --- | --- | --- | --- | --- | --- |
| 637  (Red) | 140 | CW | R780 | | 750 | | 780/60 | | APC-eFluor780 | | |  |
|  |  |  | R710 | | 690 | | 730/45 | | R718 | | |  |
|  |  |  | R660 | | 655 | | 670/30 | | AF647 | | |  |
| 561  (Yellow-Green) | 200 | CW | G780 | | 750 | | 780/60 | | PE-Cy7 | | |  |
|  |  |  | G710 | | 690 | | 710/50 | | PE-Cy5.5 | | |  |
|  |  |  | G660 | | 635 | | 670/30 | | PE-Cy5 | | |  |
|  |  |  | G610 | | 600 | | 610/20 | | PE-CF594 | | |  |
|  |  |  | G575 | | 570 | | 586/15 | | PE | | |  |
| 488  (Blue) | 200 | CW | B780 | | 770 | | 810/40 | | BB790 | | |  |
|  |  |  | B750 | | 735 | | 750/30 | |  | | |  |
|  |  |  | B710 | | 685 | | 710/50 | | BB700 | | |  |
|  |  |  | B660 | | 635 | | 670/30 | | BB660 | | |  |
|  |  |  | B610 | | 600 | | 610/20 | | BB630 | | |  |
|  |  |  | B515 | | 505 | | 530/30 | | BB515 | | |  |
| 405  (Violet) | 200 | CW | V780 | | 770 | | 810/40 | | BV785 | | |  |
|  |  |  | V750 | | 735 | | 750/30 | | BV750 | | |  |
|  |  |  | V710 | | 685 | | 710/50 | | BV711 | | |  |
|  |  |  | V655 | | 635 | | 677/20 | | BV650 | | |  |
|  |  |  | V610 | | 595 | | 605/40 | | BV605 | | |  |
|  |  |  | V570 | | 550 | | 586/15 | | BV570 | | |  |
|  |  |  | V510 | | 505 | | 525/50 | | BV510 | | |  |
|  |  |  | V450 | | 410 | | 431/28 | | BV421 | | |  |
| 355 (Ultraviolet) | 100 | CW | U780 | | 770 | | 810/40 | | BUV805 | | |  |
|  |  |  | U730 | | 690 | | 735/30 | | BUV737 | | |  |
|  |  |  | U660 | | 630 | | 670/20 | | BUV661 | | |  |
|  |  |  | U600 | | 600 | | 610/20 | |  | | |  |
|  |  |  | U570 | | 550 | | 580/20 | | BUV563 | | |  |
|  |  |  | U500 | | 490 | | 515/30 | | BUV496 | | |  |
|  |  |  | U450 | | 410 | | 450/50 | |  | | |  |
|  |  |  | U395 | | 370 | | 379/28 | | BUV395 | | |  |
|  |  |  |  |  | |  | |  | |  |  | |

Abbreviations: CW = Continuous Wave.

**BD Fortessa cytometer C**

| **Laser**  **Wavelength (nm)** | **Laser Power (mW)** | **Laser Type** | **Detector** | **Dichroic LP Filter (nm)** | **Band Pass (nm)** | **Fluorochrome** |  | |
| --- | --- | --- | --- | --- | --- | --- | --- | --- |
| 640  (Red) | 40 | DPSS | R660 | - | 660/20 |  | |  |
|  |  |  | R710 | 685 | 710/50 |  | |  |
|  |  |  | R780 | 740 | 780/60 |  | |  |
| 532  (Green) | 100 | CW | G575 | - | 575/26 | PE | |  |
|  |  |  | G610 | 600 | 610/20 | PE-CF594 | |  |
|  |  |  | G660 | 640 | 660/40 |  | |  |
|  |  |  | G710 | 690 | 710/50 |  | |  |
|  |  |  | G780 | 740 | 780/40 |  | |  |
| 488  (Blue) | 75 | CW | SSC | - | 488/10 |  | |  |
|  |  |  | B515 | 505 | 515/20 | BB515 | |  |
|  |  |  | B710 | 685 | 710/50 |  | |  |
| 405  (Violet) | 75 | DPSS | V450 | - | 450/50 | BV421 | |  |
|  |  |  | V510 | 505 | 515/20 | BV510 | |  |
|  |  |  | V560 | 557 | 560/40 |  | |  |
|  |  |  | V570 | 570 | 585/42 |  | |  |
|  |  |  | V610 | 595 | 610/20 | BV605 | |  |
|  |  |  | V655 | 630 | 660/20 |  | |  |
|  |  |  | V710 | 670 | 705/70 |  | |  |
|  |  |  | V780 | 740 | 780/60 |  | |  |

**Online Table 2. Reagents used in OMIP-0XX**

List of reagents selected in the final panel including clone, manufacturer and titer used.

| **Specificity** | **Fluorochrome** | **Clone** | **Manufacturer** | **Cat #** | **[Stock]** | **Titer**  **(xμL/100 μL)** |
| --- | --- | --- | --- | --- | --- | --- |
| γδ TCR | BUV395 | B1 | BD | 564155 | 200µg/mL | 2* |
| CD3 | BUV496 | UCHT-1 | BD | 612940 | 200µg/mL | 0.15 |
| HLA-DR | BUV563 | G46-6 | BD | 748340 | 200µg/mL | 0.6 |
| CD11c | BUV661 | B-ly6 | BD | 612967 | NA | 0.15 |
| IgD | BUV737 | IA6-2 | BD | 612798 | 100µg/mL | 0.3 |
| CD27 | BUV805 | M-T271 | BD | 742012 | 200µg/mL | 1.2* |
| Perforin | BV421 | γG9 | BD | 563393 | 200µg/mL | 0.6 |
| CD4 | BV480 | SK3 | BD | 566104 | 50µg/mL | 0.3 |
| CD8 | BV570 | RPA-T8 | BioLegend | 301038 | 100µg/mL | 0.6 |
| CD16 | BV605 | 3G8 | BD | 563172 | 600µg/mL | 2 |
| CD161 | BV650 | DX12 | BD | 563864 | NA | 0.3 |
| CD56 | BV711 | HCD56 | BioLegend | 318336 | 100µg/mL | 0.6 |
| CD66 | BV750 | B1.1 | BD | 746904 | 200µg/mL | 0.15 |
| CD45 | BV785 | HI30 | BioLegend | 304048 | 50µg/mL | 0.3 |
| CD57 | BB515 | NK-1 | BD | 565285 | 150µg/mL | 0.01 |
| CD32 | BB630 | FLI8.26 | BD | CUSTOM | 200µg/mL | 0.3 |
| CD14 | BB660 | MφP9 | BD | CUSTOM | 200µg/mL | 0.15 |
| CD38 | BB700 | HIT2 | BD | 566445 | 200µg/mL | 0.6 |
| Vδ2 | BB790 | B6 | BD | CUSTOM | 200µg/mL | 0.3 |
| TRAV1.2 | PE | 3C10 | BioLegend | 351706 | 100µg/mL | 0.6 |
| CCR7 | PE-CF594 | 150503 | BD | 562381 | 50µg/mL | 1.2* |
| CD33 | PE-Cy5 | WM53 | BD | 551377 | 13µg/mL | 0.2 |
| CD127 | PE-Cy5.5 | eBioRD5 | eBioscience | 35-1278-42 | 25µg/mL | 0.3 |
| Ki67 | PE-Cy7 | B56 | BD | 561283 | NA | 0.15 |
| Granzyme B | AF647 | GB11 | BioLegend | 515406 | 50 μg/mL | 2 |
| CD19 | R718 | HIB19 | BD | 567343 | 200µg/mL | 1 |
| CD45RA | APC-eFluor 780 | HI100 | eBioscience | 47-0458-42 | 250µg/mL | 0.15 |

***** γδ TCR, CD27 and CCR7 are stained first in a final volume of 50μL, all other antibodies are added during the second staining step in a final volume of 100μL.

**Online Table 3. Priority levels of reagent combinations**

| **Priority level** | **Category / Rationale** | **Reagents** |
| --- | --- | --- |
| 1 | Lineage markers for major cell subset identification | CD45, CD66, CD14, CD33, CD3, CD4, CD8, CD19, CD56, CD16, TRAV1.2, γδ TCR, Vδ2 |
| 2 | Cell subset differentiation status  / activation | CCR7, CD45RA, CD57, IgD, CD27, HLA-DR, CD127, CD161, CD38 |
| 3 | Cytolytic function  (T and NK cells) | Perforin, Granzyme B |
| 4 | Proliferation | Ki67 |
| 5 | Myeloid cell subset | CD11c |
| 6 | Fc receptor | CD32 |

**Online Table 4. Spill-over spreading error (SSE) matrix (SSM) generated with compensation beads using the reagents from panel (online table 2).**

**Online Table 5. Antibody titrations**

| **Specificity** | **Fluorochrome** | **Titrated in association with** | **Co-stain antibodies** | **Gate** |
| --- | --- | --- | --- | --- |
| γδ TCR | BUV395 | CD66 BV750 | Vδ2 BB700; CD3 BUV496 | T cells |
| CD3 | BUV496 | CD33 PE-Cy5; CD45 BV785 | None | Singlets |
| HLA-DR | BUV563 | CD45RA APC-R780; CD127 PE-Cy5.5 | CD4 BV480 | Singlets |
| CD11c | BUV661 | None | CD3 BUV496 | Singlets |
| IgD | BUV737 | CD16 BV605 | None | Lymphocytes |
| CD27 | BUV805 | None | CD19 BV421; CD4 BV480; CD3 BUV496 | T cells |
| Perforin | BV421 | CD38 BB700; Ki67 PE-Cy7 | CD3 BUV496; CD16 BV605 | Lymphocytes |
| CD4 | BV480 | CD14 BB660 | None | Lymphocytes |
| CD8 | BV570 | CD56 BV711 | None | Lymphocytes |
| CD16 | BV605 | IgD BUV737 | None | Lymphocytes |
| CD161 | BV650 | TRAV1.2 PE | CD3 BUV496 | Lymphocytes |
| CD56 | BV711 | CD8 BV570, CD32 BB630 | None | Lymphocytes |
| CD66 | BV750 | γδ TCR BUV395 | None | Singlets |
| CD45 | BV785 | CD33 PE-Cy5; CD3 BUV496 | None | Singlets |
| CD57 | BB515 | Vδ2 BB790 | CD3 BUV496 | Lymphocytes |
| CD32 | BB630 | CD3 BUV496 | None | Singlets |
| CD14 | BB660 | CD4 BV480 | None | Singlets |
| CD38 | BB700 | Perforin BV421; Ki67 PE-Cy7 | None | Singlets |
| Vδ2 | BB790 | CD57 BB515 | γδ TCR BUV395, CD3 BUV496 | T cells |
| TRAV1.2 | PE | CD161 BV650 | CD3 BUV496 | Lymphocytes |
| CCR7 | PE-CF594 | CD19 R718 | CD3 BUV496; CD4 BV480; CD8 BV570; CD45RA APC-Cy7 | Lymphocytes |
| CD33 | PE-Cy5 | CD45 BV785; CD3 BUV496 | None | Singlets |
| CD127 | PE-Cy5.5 | CD45RA APC-R780; HLA-DR BUV563 | CD4 BV480 | Lymphocytes |
| Ki67 | PE-Cy7 | Perforin BV421; CD38 BB700 | CD3 BUV496 | Lymphocytes |
| Granzyme B | AF647 | None | CD8 BV570; CD56 BV711; CD16 BV650 | Lymphocytes |
| CD19 | R718 | CCR7 PE-CF594 | CD3 BUV496; CD4 BV480; CD8 BV570; CD45RA APC-Cy7 | Lymphocytes |
| CD45RA | APC-eFluor 780 | None | CD8 BV570; CD4 BV480; CCR7 PE-CF594 | Lymphocytes |

**Online Table 6. Reagents tested in OMIP-0XX but not included in the final panel**

| **Fluorochrome** | **Specificity** | **Clone** | **Manufacturer** | **Cat #** | **Reason for exclusion** |
| --- | --- | --- | --- | --- | --- |
| R718 | CCR7 | 150503 | BD | 751859 | Poor separation after fixation (WB, PBMCs) |
| BUV395 | CCR7 | 150503 | BD | 625526 | Poor separation compared to CCR7-PE-CF594, replaced by γδ TCR |
| BUV615 | CD11b | ICRF44 | BD | 751375 | Spreads into CCR7-PE-CF594 |
| BV750 | CD11b | ICRF44 | BD | 747357 | CD66 prioritization to exclude granulocytes |
| BB630 | CD127 | HIL-7R-M71 | BD | CUSTOM | Poor separation after fixation (WB, PBMCs) |
| BV605 | CD127 | A019D5 | Biolegend | 351334 | Poor separation after fixation (WB, PBMCs) |
| BV605 | CD127 | A7R34 | Invitrogen | 56-1271-82 | Poor separation after fixation (WB, PBMCs) |
| BB630 | CD161 | DX12 | BD | CUSTOM | Poor separation after fixation (WB, PBMCs) |
| BV711 | CD163 | GHI/61 | BD | 563889 | We prioritized inclusion of MAITs markers over CD163 after excluding MR1 tetramer |
| PE-CF594 | CD19 | HIB19 | BD | 562294 | CCR7 prioritization |
| BUV805 | CD20 | 2H7 | BD | 612906 | CD19 prioritization to identify plasmablasts more accurately |
| R718 | CD27 | M-T271 | BD | 567679 | Poor separation when added to the full panel |
| APC-Cy7 | CD45RA | HI100 | BD | 304128 | We experienced several tandem dissociation, tandem does not seem stable |
| PE-Cy5.5 | CD56 | CMSSB | eBioscience | 35-0567-41 | CD127 prioritization |
| BV650 | CXCR5 | RF8B27 | Biolegend | 740528 | Poor separation after fixation. Low frequency of CXCR5+ expressing CD4 T cells in blood |
| BV750 | CXCR5 | J252D4 | Biolegend | 356941 | Poor separation after fixation. Low frequency of CXCR5+ expressing CD4 T cells in blood |
| PE-Dazzle594 | CXCR5 | J252D4 | Biolegend | 356928 | Poor separation after fixation. Low frequency of CXCR5+ expressing CD4 T cells in blood |
| PE-CF594 | γδ TCR | B1 | BD | 562511 | CCR7 prioritization |
| BUV737 | IgG | G18-175 | BD | 564861 | No staining after fixation (WB, PBMCs) |
| PE | IgG | M1310G05 | Biolegend | 410707 | No staining after fixation (WB, PBMCs) |
| PE | IgG1 | HP6001 | Southern Biotech | Sep-54 | No staining after fixation (WB, PBMCs) |
| PE | IgM | MHM-88 | Biolegend | 314507 | TRAV1.2 prioritization |
| APC | Granulysin | DH2 | BD | 348010 | Poor staining |
| BB790 | NKG2C | 134591 | BD | CUSTOM | Lot-to-lot variability |
| BV650 | NKG2C | 134591 | BD | 624486 | CD161 prioritization |
| BB630/BB700 | PD-1 | EH12.1 | BD | CUSTOM | Good separation. PD-1 was initially tested to identify Thf cells that co-express CXCR5. Since CXCR5 staining resulted in poor separation, PD-1 was excluded from the panel |
| PE-Cy7 | PD-1 | EH12.2H7 | Biolegend | 329918 | Good separation. PD-1 was initially tested to identify Thf cells that co-express CXCR5. Since CXCR5 staining resulted in poor separation, PD-1 was excluded from the panel |
| PE | CD1d:PBS57 tetramer | - | NIH Core facility | - | Poor separation after fixation |
| PE | MR1:5-OP-RU tetramer | - | NIH Core facility | - | Unspecific staining on CD3- T cells. Percentage of MR1 tetramer+ cells amongst TRAV1.2+ CD161+ cells was highly variable across donors. MR1-tetramer was then replaced by phenotypic markers to identify MAITs |
| BUV570 | Vα24Jα18 | 6B11 | BD | 748830 | No staining after fixation (WB, PBMCs) |
| BV650 | Vδ2 | B6 | BD | 743752 | CD161 prioritization |
| - | Fc block | - | BD | 564219 | No improvement of staining performance or reduction of non-specific staining (which was not observed even without Fc block) |

**Online Figure 1. Antibody titrations**

Two-fold serial dilutions of antibodies were tested and samples were acquired using BD FACSymphony A5 cytometer B. The starting volume is indicated on the x-axis for each titration. Since the markers in our panel are expressed by different cell types and subsets at different levels, the parent gate may differ (see **Online Table 5**). Markers that are expressed on multiple cell types (e.g HLA-DR, CD16) were assessed on all relevant populations. Files of each titration experiment were concatenated. Red rectangles indicate the chosen titer based on the staining index calculation, spread into other channels and frequencies of positive cells. Final titration results are expressed as µL/50 or 100µL of staining cocktail (see **Online Table 2**).

**Online Figure 2. Evaluation of fluorochrome combinations with high values in SSM**

Fluorochrome excited by yellow-green (A), violet (B), UV (C) and blue (D) laser with fluorochrome combinations with values ≥ 3.51 in the SSM are depicted. Fluorochrome on the y-axis generates spread into the channel on the x-axis. Plots are gated on either singlets, CD45+ cells or lymphocytes, depending on the most relevant gate for which marker combination is assessed. Full panel and FMO controls are shown. Samples were acquired using BD FACSymphony A5 cytometer B.

**Online Figure 3. Impact of fixation on anti-CD127 clones**

(A) PBMCs from three healthy donors were stained with anti-CD3, anti-CD27 and different clones of anti-CD127 labelled with different fluorochromes before or after fixation in different experiments. While fixation interferes with CD127 staining of clones A019D5 and HIL-7R-M71, 35-1278-41 remained unaffected. Samples were acquired using BD FACSymphony A5 cytometer A (clones HIL-7R-M71 and 35-1278-41) and BD Fortessa cytometer C (clone A019D5 ) (B) Titration of anti-CD127 clone 35-1278-41 labelled with PE-Cy5.5 on fixed WB.

**Online Figure 4. Fixative interferes with CXCR5 staining**

(A) PBMCs from one healthy donor were stained with anti-CXCR5 antibody (clone J252D4) labelled with PE-CF594 before or after fixation. PBMCs were co-stained with anti-CD3, anti-CD4 and anti-CD19 to detect CXCR5 expression on CD4 T cells and B cells, respectively. Histograms show CXCR5 expression on fresh (green) and fixed (red) CD3+ T cells (top panel) and non-T cell lymphocytes (bottom panel) using FMO controls. Samples were acquired using BD Fortessa cytometer C. (B) Titrations of anti-CXCR5 antibody (clone J252D4) labelled with PE-CF594 (top panel) or BV750 (bottom panel) on fixed WB from two independent healthy donors. Samples were acquired using BD FACSymphony A5 cytometer A. (C) Files of each titration experiments shown in (B) were concatenated. WB was co-stained with anti-CD4 and anti-CD19 or anti-CD20 to detect CXCR5 expression on CD4 T cells and B cells, respectively. T cells (black) are overlaid on non-T cell lymphocytes (blue). Good resolution was achieved for B cells, whereas no signal was detected for CD4 T cells.

**Online Figure 5. Fixative interferes with Vα24Jα18 TCR staining**

PBMC were stained with a small panel including anti-CD3, anti-CD161 and anti-CD56 to assess Vα24Jα18 expression on invariant NKT cells prior and after fixation using FMO controls. Vα24Jα18 staining was not successful on fixed cells. Samples were acquired using BD FACSymphony A5 cytometer A.

**Online Figure 6. Fixative interferes with IgG but not IgM or IgD staining**

(A) Titrations of anti-IgG antibody (clone M1310G05; top panel) and anti-IgG1 (clone HP6001; bottom panel) conjugated with PE on fixed WB from two independent healthy donors do not allow resolution of IgG signal. Samples were acquired using BD FACSymphony A5 cytometer A. (B) PBMCs and fixed WB from the same healthy donor were stained with anti-IgD labelled with BB515 and either anti-IgG (clone M1310G05) or anti-IgG1 (clone HP6001). Samples were acquired using BD Fortessa cytometer C. (C) anti-IgM labelled with PE was tested on fresh and fixed PBMCs. Cells were co-stained with anti-CD19 or anti-CD20 to allow detection of IgG, IgM and IgD on B cells. Samples were acquired using BD FACSymphony A5 cytometer B.

**Online Figure 7. Comparison of anti-CCR7 labelled with different fluorochromes**

PBMCs and fixed WB from one healthy donor were stained at 37ºC with anti-CD4 anti-CD45RA and anti-CCR7 (clone 150503) labelled with R718, PE-CF594 or BUV395 before or after fixation. All antibodies were titrated on fixed WB and files of each titration experiments were concatenated. Samples were acquired using BD FACSymphony A5 cytometer B.

**Online Figure 8. Optimizing staining resolution of anti-γδ TCR**

(A) Fixed WB from two healthy donors was stained with anti-γδ TCR antibody labelled with PE-CF594 (clone B1) by adding the antibody either in the full antibody mix or separately at 37ºC (“alone”). (B) Comparison of anti-γδ TCR-specific clone 11F2 (labelled with BB515) with B1 (labelled with PE-CF594) on fixed WB from one healthy donor. WB was co-stained with anti-CD3 BUV515 and anti-Vδ2 BB780. B1 clone may improve identification of Vδ2-negative γδ T cells while γδ T cells co-expressing Vδ2 may not be affected by the clone selection. Samples were acquired using BD FACSymphony A5 cytometer B.

**Online Figure 9. Illustration of hierarchical gating rationale and cell subset identification and characterization**

Schematic of the hierarchical marker selection/gating and assessment of different cell lineages and subsets.

**Bibliography**

1. Imbratta C, Gela A, Bilek N, Mabwe S, Cloete Y, Mortensen R, et al. Qualification of the differential leukocyte count and immunophenotyping in cryopreserved ex vivo whole blood assay. Cytometry A. 2023.

2. Park LM, Lannigan J, Jaimes MC. OMIP-069: Forty-Color Full Spectrum Flow Cytometry Panel for Deep Immunophenotyping of Major Cell Subsets in Human Peripheral Blood. Cytometry A. 2020;97(10):1044-51.

3. Nemes E, Kagina BM, Smit E, Africa H, Steyn M, Hanekom WA, et al. Differential leukocyte counting and immunophenotyping in cryopreserved ex vivo whole blood. Cytometry A. 2015;87(2):157-65.
